# Thermo‐Responsive Self‐Recoverable Porous Sensors with Writable Electrodes: Advancing Wearable Motion Detection

**DOI:** 10.1002/advs.202517254

**Published:** 2025-12-16

**Authors:** Ying Gao, Xingyi Dai, Yuanyuan Zhang, Guohao Fang, Gui Li, Yu Zheng, Long‐Biao Huang, Biqin Dong

**Affiliations:** ^1^ School of Environment and Civil Engineering Guangdong Provincial Key Laboratory of Intelligent Disaster Prevention and Emergency Technologies for Urban Lifeline Engineering Dongguan University of Technology Dongguan 523808 P. R. China; ^2^ College of Civil and Transportation Engineering Guangdong Province Key Laboratory of Durability for Marine Civil Engineering The Key Laboratory on Durability of Civil Engineering in Shenzhen Shenzhen University Shenzhen 518060 P. R. China; ^3^ Key Laboratory of Optoelectronic Devices and Systems of Ministry of Education and Guangdong Province College of Physics and Optoelectronic Engineering Shenzhen University Shenzhen 518060 P. R. China; ^4^ National Key Laboratory of Green and Long‐Life Road Engineering in Extreme Environment Shenzhen University Shenzhen 518060 P. R. China; ^5^ Institute for Advanced Study Shenzhen University Shenzhen 518060 P. R. China

**Keywords:** Dual‐modes, Human motion detection, Self‐recovering, Shape memory polymer, Shape‐editability

## Abstract

The remarkable applications of flexible and wearable sensors in the fields of human motion detection and health monitoring have attracted extensive attention from academic and industrial pools. However, creating advanced sensors capable of self‐recovery, shape‐editability, and multi‐mode for long‐term motion detection still remains challenging. Herein, a self‐recoverable flexible porous sensor with diverse designability of electrodes is developed for comprehensive motion detection. Shape memory polymer (SMP) is utilized as a core material to manufacture the porous electrodes, which endows the sensor with excellent self‐recoverability, enhanced structural robustness, and high flexibility. The porous electrodes further enable dual triboelectric and piezoresistive sensing modes, which can achieve versatile detection capabilities for various body movements and motion patterns. A writable vapor phase polymerization technique is employed to realize the shape‐editability of the electrodes, ensuring adaptability to diverse body shapes and complex movements. The novel sensors are integrated into smart insoles, joint monitoring bands, and intelligent gloves, demonstrating the capabilities of the sensors in plantar pressure monitoring, joint motion tracking, tactile sensing, and information interaction. This work offers a promising strategy for the development of advanced wearable sensors with improved sustainability, reliability, and conformability.

## Graphical abstract

A self‐recoverable flexible porous sensor with diverse designability of electrodes is developed through writable vapor phase polymerization using shape memory polymers (SMPs) as the fundamental materials. The sensors enable long‐term comprehensive human motion detection. This work contributes to a self‐recoverable flexible sensing technology for next‐generation wearable devices, human‐machine interfaces, and robotics.

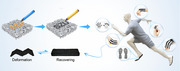

## 1 Introduction

Wearable sensors have garnered widespread attention from the academic and industrial pools due to their diverse practical applications, such as artificial electronic skins, human‐machine interfaces, soft robotics, human motion detection, health monitoring, etc.^[^ ^1^, ^2^, ^3^, ^4^ ^]^ For the sake of human motion detection, wearable sensors must satisfy the requirements, such as long‐term durability, flexibility, morphological adaptability, lightweight, and multimodal capabilities.^[^ ^5^, ^6^, ^7^ ^]^ Over the past decade, various sensors have been successfully developed and applied to the human motion detection of individual parts of the human body.^[^ ^8^, ^9^, ^10^ ^]^ With the increasing concern about life quality and human health, comprehensive human motion detection is being pursued. However, the complex contours and diverse motion patterns of the human body pose challenges for current sensors in detecting motion across its various elements. Consequently, there is an urgent need to develop advanced sensors that can incorporate these essential features and thereby achieve comprehensive human motion monitoring.

Compared to traditional rigid sensors, flexible sensors adapt better to the contours of the human body, enabling complex motion monitoring.^[^ ^11^ ^]^ Xu et al. developed a portable and wearable flexible sensor system that integrates pressure and strain sensors. This system can be attached to the throat to detect high‐frequency vocal vibrations and low‐frequency muscular movements, thereby enhancing the rehabilitation for stroke‐induced aphasia.^[^ ^12^ ^]^ Yang et al. reported a dual‐mechanism flexible iontronic pressure sensor (FIPS) with high sensitivity and full‐range linearity. When integrated into a smart insole, the sensor enables gait recognition, thereby allowing early prediction of fracture risk.^[^ ^13^ ^]^ Zhou et al. introduced a flexible 3D‐printed strain sensor. The sensor can precisely monitor joint motions by attaching to the fingers, wrists, elbows, neck, and knees.^[^ ^11^ ^]^ These advances demonstrate how flexible sensors can significantly improve the quality and accessibility of healthcare. Correspondingly, numerous materials have been developed to fabricate such flexible sensors, such as polymer elastomers, porous materials, and hydrogels.^[^ ^14^ ^]^ Porous materials with high flexibility, high porosity, low density, and large specific surface area have emerged as promising candidates for flexible active sensing layers.^[^ ^15^, ^16^, ^17^, ^18^, ^19^ ^]^ He et al. developed a strain sensor based on porous CNT‐GR/PDMS nanocomposites, which can conform to the finger, wrist, elbow, and knee to respond to their diverse bending degrees.^[^ ^20^ ^]^ Chen et al. presented a flexible pressure sensor with a porous structure, which can also detect human motion, including finger bending, wrist bending, elbow bending, and knee bending.^[^ ^21^ ^]^ Kim et al. reported a PDMS‐covered porous network sponge, and a sensor derived from the sponge was effectively used to simultaneously monitor small and large deformations of the human body.^[^ ^22^ ^]^ Yang et al. revealed a flexible sensor with hierarchical porous structures, which can achieve bending measurement at different angles by attaching to the elbow joint of the human body.^[^ ^23^ ^]^ Wang et al. presented a breathable and hydrophobic pressure sensor with high sensitivity and a wide detection range based on a porous ionic electrolyte and a nanofiber, which can monitor the human body's walking, running, jumping, and falling states by attaching to the knee joint.^[^ ^24^ ^]^ Thus, preparing materials into a porous structure can be effective in enhancing flexibility and achieving complex human motion detection.

However, most sensors are inherently susceptible to degradation under repeated stress, which compromises their longevity and reliability.^[^ ^25^ ^]^ Although repair strategies can temporarily mitigate damage, the underlying structural deterioration has not been addressed yet.^[^ ^26^ ^]^ Shape memory polymers (SMPs) are stimulus‐responsive materials that have a strong capability to maintain a temporary shape and recover initial or programmed shapes under an external stimulus, such as heat, light, and magnetic fields.^[^ ^27^ ^]^ Thus, SMPs had presented a potential solution by enabling self‐recovery, thereby enhancing sensor sustainability.^[^ ^28^, ^29^, ^30^, ^31^ ^]^ Lee et al. demonstrated a wearable device based on SMPs, which had recovery capability to the micropatterns by heating at 55 °C.^[^ ^26^ ^]^ Huang et al. used SMPs as fundamental materials to fabricate wearable sensors and thus effectively improved their performance, recoverability, and structural robustness.^[^ ^32^ ^]^ Wang et al. developed self‐powered motion detection sensors based on SMP, and the sensor exhibited excellent durability during monitoring various actions such as pressing, bending, and torsion.^[^ ^33^ ^]^

Furthermore, the suitable shape of sensors is equally crucial for conforming to dynamic human body shapes.^[^ ^34^, ^35^ ^]^ The shapes of reported sensors are simple and fixed, which limits the adaptability for diverse body shapes and complex kinematics.^[^ ^11^, ^36^, ^37^, ^38^ ^]^ These limitations underscore the demand for sensors with arbitrary shapes.^[^ ^39^ ^]^ Thereby, realizing the shape‐editability of sensors can further enhance their adaptability. Moreover, the varying structure and motion patterns of different human body parts pose significant challenges for complex motion monitoring with single‐mode sensing.^[^ ^40^ ^]^ Developing sensors with multi‐mode sensing shows a promising way to achieve comprehensive motion detection.^[^ ^23^ ^]^ However, the integration of multimodal sensing capabilities into the same sensing layer remains challenging.^[^ ^41^ ^]^ Recently, the conductive porous material has demonstrated the capability of multiple sensing modes.^[^ ^15^, ^23^ ^]^ Thus, high‐performance comprehensive human motion detection can be realized by developing advanced multimodal sensors that use conductive porous materials with flexibility, self‐recoverability, and shape‐editability as the electrodes.

In this work, a self‐recoverable flexible porous sensor with diverse designability of electrodes was developed to achieve comprehensive motion detection. A highly conductive SMP sponge was developed for the fabrication of porous sensors via a salt sacrificial template method, followed by vapor phase polymerization of a conductive polymer. Based on the fabrication mechanism, the shape‐editability of the electrodes was achieved through stencil‐ and writing‐based techniques, which enhanced the adaptability of sensors to the human body. Benefiting from the self‐recoverability of the SMP, the prepared sensors can recover their original structure and performance upon thermal stimulation. Moreover, as the sensor can operate in dual triboelectric and piezoresistive sensing modes, the novel flexible sensor has demonstrated versatile applications, including smart insoles for plantar pressure mapping, joint bands for movement monitoring, and intelligent gloves for materials recognition, palm pressure mapping, Morse code simulation, and rehabilitation tracking. Overall, this work provides a novel strategy for designing and preparing sustainable, reliable, and conformable wearable sensors, which have shown great practicability in human motion detection, health conditions monitoring, medical diagnosis, human‐machine interfaces, and artificial intelligence.

## 2 Results and Discussion

### 2.1 Synthesis and Characterization of the EVA@PPy Sponge

**Figure 1:**
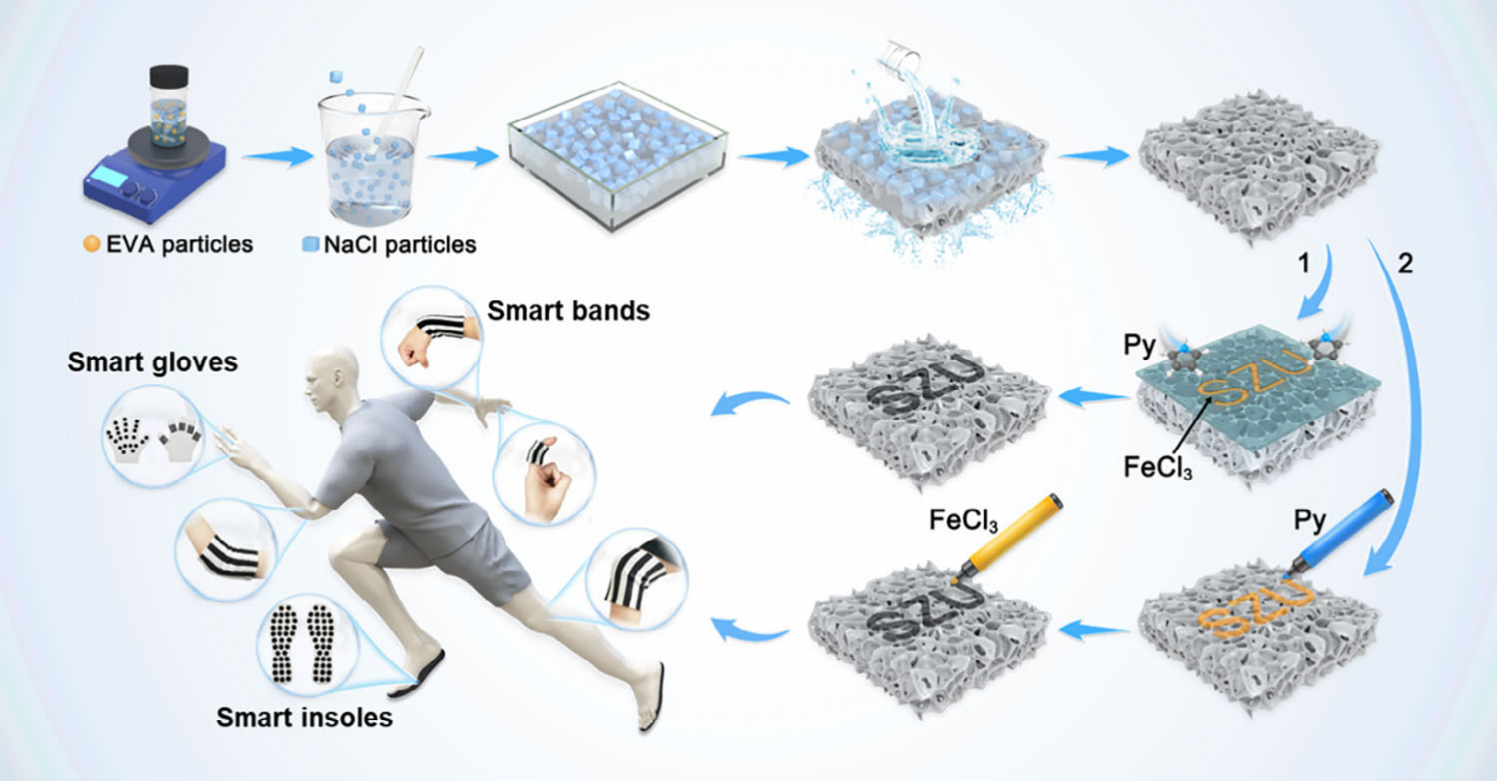
Schematic of the fabrication process of the EVA@PPy sponges and their applications.

The schematic illustration of the synthesis process and applications of the EVA@PPy is presented in **Figure** **1**. As seen, the EVA@PPy sponge is synthesized through a two‐step process involving porous EVA matrix formation using a salt sacrifice template technique, followed by vapor phase polymerization of PPy. As the vapor‐phase polymerization of PPy is confined to catalyst‐containing regions, two distinct methods, including a stencil‐based approach and a freehand drawing technique, are applied to achieve customized PPy patterns on the EVA sponge. For the stencil method, an SZU‐patterned mask was positioned on the EVA sponge surface. The exposed SZU regions are impregnated with FeCl_3_ catalyst solution. Then the entire assembly is transferred to a vacuum desiccator containing pyrrole monomer to facilitate the vapor‐phase polymerization of PPy, which precisely produces the designed SZU pattern. For the freehand drawing approach, the SZU pattern is drawn on the sponge using a marker pen preloaded with pyrrole solution and an FeCl_3_‐filled marker pen in sequence, allowing for complete customization of the electrode geometry. Accordingly, the shape‐editable electrodes are employed to produce various wearable devices, such as smart insoles, smart gloves, and smart bands, which allow for comprehensive human motion detection.

**Figure 2:**
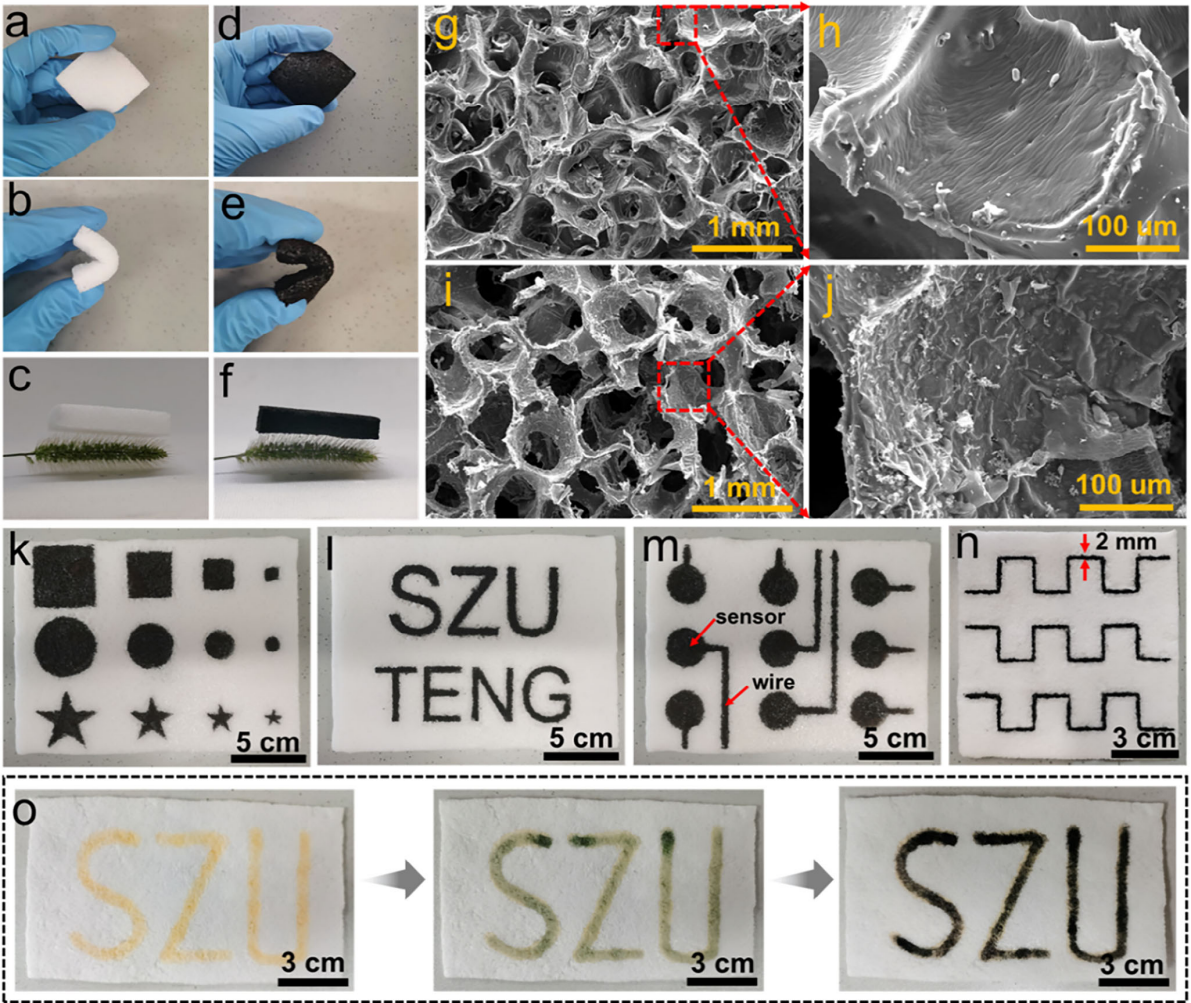
Characterization of the prepared EVA@PPy sponge. a,b) Photographs of the EVA sponge in normal and bending states. c) The ultralight EVA sponge supported by *Setaria viridis*. d,e) Photographs of the EVA@PPy sponge in normal and bending states. f) The ultralight EVA@PPy sponge supported by *Setaria viridis*. g,h) SEM images of the microstructure and pore walls of the EVA sponge. i,j) SEM images of microstructure and pore walls of the EVA@PPy sponge. k–n) Photographs of the EVA sponge with various PPy patterns fabricated by a stencil‐based approach. o) Photographs of an SZU pattern on the EVA sponge fabricated by the freehand drawing technique.

The as‐prepared EVA sponge demonstrates excellent flexibility and bendability, as shown in **Figure** **2**a,b. It can be supported on the vertical microstructure of *Setaria viridis*, indicating its light‐weight feature (Figure 2c). The PPy is integrated with the EVA sponge framework after the vapor phase polymerization of the PPy, and the obtained conductive EVA@PPy sponge shows a black appearance. The integration process does not compromise the sponge's flexibility, bendability, and lightweight properties (Figure 2d–f), showing the potential of the sponge in developing flexible ultra‐lightweight sensors. In addition, the SEM images reveal the porous nature of the EVA sponge with pore diameters of ≈0.5 mm and smooth pore walls (Figure 2g,h). The pore structure of the sponge has not been damaged after the vapor phase polymerization of PPy, indicating that the introduction of PPy does not make a negative difference to the pore structure (Figure 2i). A wrinkled PPy film is observed on the surface of pore walls (Figure 2j), confirming the successful and uniform integration of the PPy into the EVA sponge. Furthermore, the porous structure ensures high moisture permeability, as evidenced by Figure S2 (Supporting Information), thus laying the foundation for long‐term wearing comfort.^[^ ^24^ ^]^

To confirm the in situ polymerization of the PPy on the EVA sponge frameworks, the chemical structure of the EVA and EVA@PPy sponges is investigated through FTIR. As displayed in Figure S3 (Supporting Information), four characteristic peaks, including the stretching vibrations of CH_3_ (2916 cm^−1^), CH_2_ (2848 cm^−1^), C═O (1737 cm^−1^), and C‐O‐C (1237 cm^−1^), can be observed in the FTIR spectrum of the EVA.^[^ ^42^ ^]^ In the spectrum of the EVA@PPy, both the characteristic peaks of the EVA and the unique peaks of the PPy at N‐H stretch (3359 cm^−1^), C═C stretch (1548 cm^−1^), C‐N stretch (1305 cm^−1^) and C‐H stretch (1040 cm^−1^) are observed,^[^ ^43^ ^]^ which indicates that the PPy layer is successfully coated on the EVA sponge frameworks.

Benefiting from the vapor phase polymerization of the PPy, sensors with various shapes and sizes can be precisely designed (Figure 2k,l), indicating excellent controllability, flexibility, and scalability in creating intricate geometries for diverse applications. Also, wires used to integrate with the sensors can be formed directly, and the width of wires can be precisely controlled to achieve thin configurations, see Figure 2m,n. The line width can be further reduced to 0.5 mm, as shown in Figure S4a (Supporting Information). Additionally, the measured resistances reveal a correlation factor above 0.9 for lines (Figure S4b, Supporting Information), confirming excellent uniformity across different line widths. The uniformity is also quantitatively assessed through the minimum feasible side length achievable for various patterns (Figure S4c, Supporting Information). In addition, the PPy layer can be selectively formed on one of the EVA sponge surfaces (Figure S5, Supporting Information), which enables the detection of sensing signals from one side of the sponges. This can minimize the influence of noise and thereby enhance sensing accuracy. Moreover, as displayed in Figure 2o and Video S1 (Supporting Information), the flexibility and versatility of the freehand drawing technique employed to customizable pattern fabrication further enhance the shape‐editability of the sensors.

### 2.2 Shape Memory Effect and Conductivity of the EVA@PPy Sponge

The shape memory effect of the EVA sponge is investigated. As seen in Figure S6 (Supporting Information), the cuboid EVA sponge specimen is heated to 65 °C and subsequently compressed to an M‐shape configuration. The EVA sponge keeps the M‐shape at room temperature. Upon reheating from room temperature to 65 °C, the M‐shape specimen reverts to its original cuboid form in 5 s, which confirms the rapid self‐recovery capability of the EVA sponge under thermal stimulus. In addition, the SEM images display the microstructure change of the EVA sponge during the deformation‐recovery process. The internal pore structure collapses during deformation and fully recovers to its original state as the EVA sponge returns to its initial shape, which proves the self‐recoverability of the EVA sponge microstructure.

**Figure 3:**
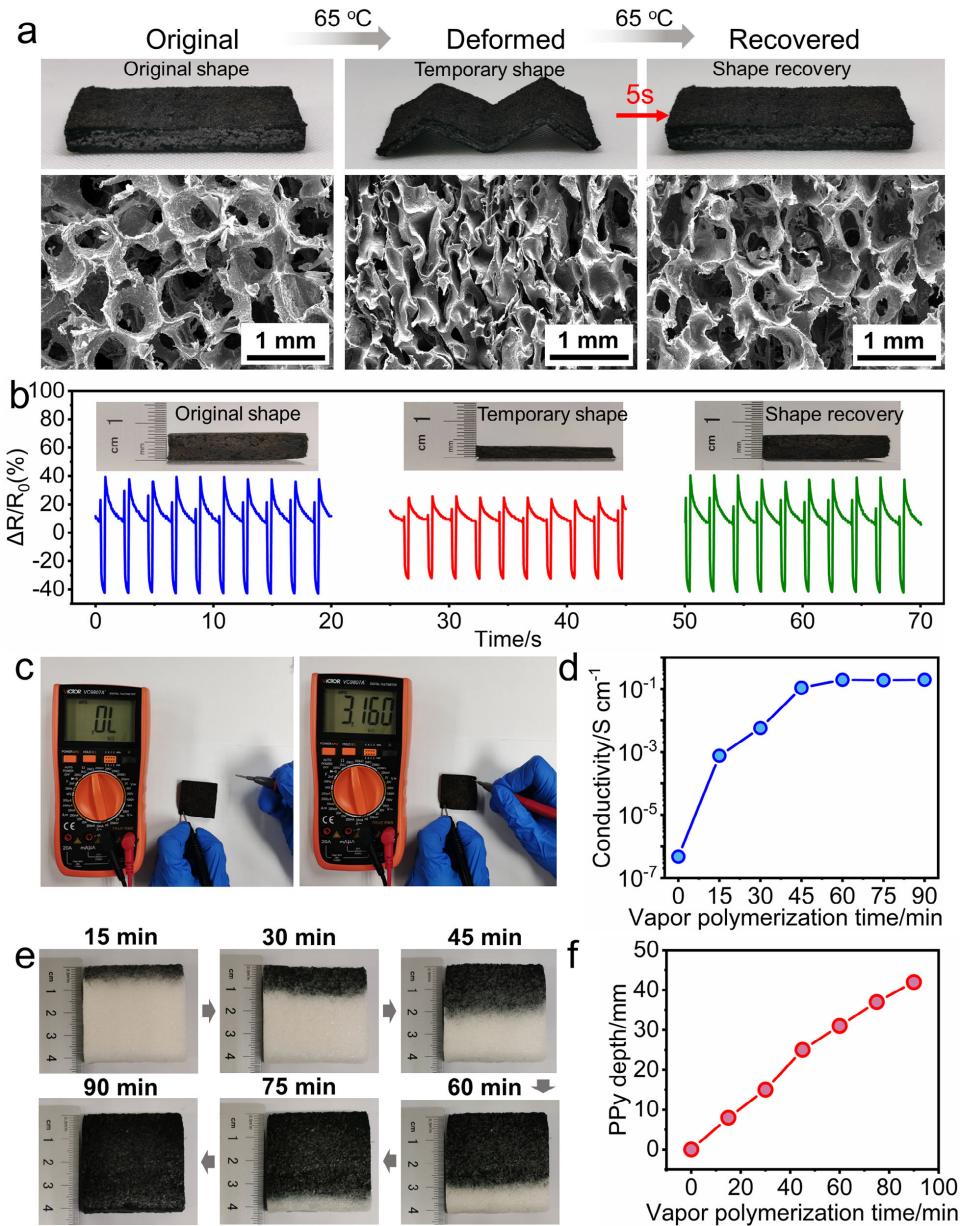
Shape memory effect and conductivity of the EVA@PPy sponge. a) Macroscopic photographs and SEM micrographs of the EVA@PPy sponge during the deformation‐recovery process. b) Relative resistance changes of the EVA@PPy‐based piezoresistive sensor during the deformation‐recovery process. c) Conductivity of the EVA@PPy sponge. d) The linear relationship between conductivity and vapor phase polymerization times of PPy. e,f) The penetration depth of PPy in the EVA sponge at different vapor phase polymerization durations.

The EVA@PPy sponge also shows the rapid self‐recovery capability as the EVA sponge (**Figure** **3**a), indicating that the vapor phase polymerization of PPy does not compromise this critical property. In addition, the relative resistance (*ΔR*/*R_0_*) of the EVA@PPy sponge‐based piezoresistive sensor decreases and restores to its original value during the deformation‐recovery process (Figure 3b). Furthermore, the open‐circuit voltage (*V_oc_*) of the EVA@PPy sponge‐based TENG increases during deformation, while it decreases to the original value after the recovery of the sponge (Figure S7, Supporting Information). The increase in *V_oc_* is attributed to pore collapse and enhanced conductivity. The recovery of the EVA@PPy sponge structure and the EVA@PPy‐based sensor performance demonstrate the self‐recoverability of EVA@PPy sponge, significantly enhancing the durability of the sensor and extending its operational lifetime.

The incorporation of PPy endows the EVA matrix with conductivity, as shown in Figure 3c. The conductivity of the EVA@PPy sponge gradually increases with the vapor phase polymerization time of PPy, see Figure 3d. And the conductivity of a sample (38 mm × 38 mm × 7 mm) reaches its maximum value at 60 min, with no further increase upon prolonged polymerization, indicating that the integration of PPy saturates at 60 min. In addition, the influence of the polymerization time on the depth of PPy integration is investigated. As shown in Figure 3e,f, the depth of PPy integration increases with polymerization time, exhibiting a linear relationship. This provides guidance for selecting the optimal polymerization time to fabricate the EVA@PPy‐based sensors with varying thickness.

### 2.3 Triboelectric Performance of the EVA@PPy Sponge

**Figure 4:**
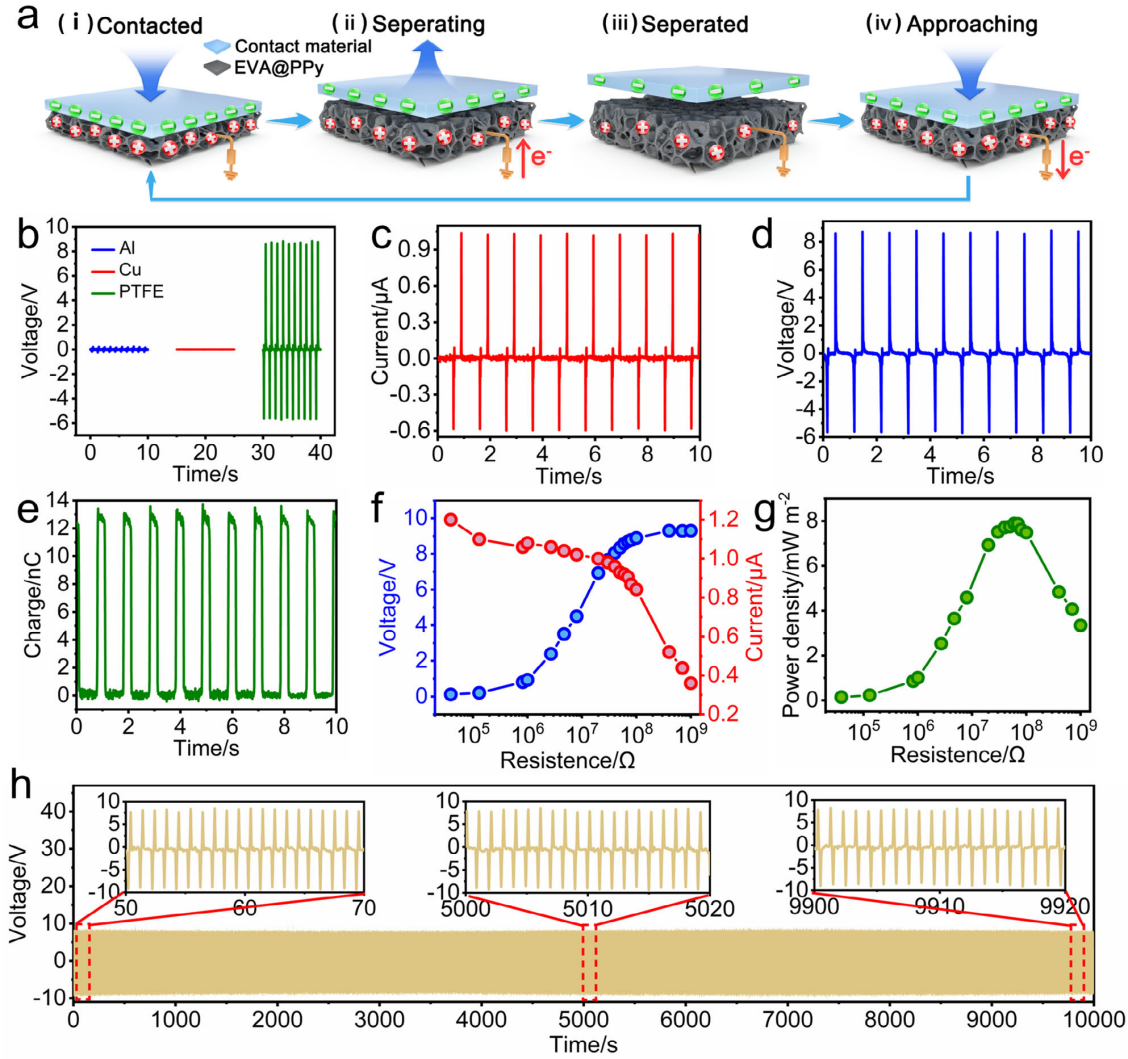
Triboelectric performance of the EVA@PPy sponge. a) Schematic of the working mechanism of the EVA@PPy‐based TENG. b) The *V_oc_* values of the EVA@PPy‐based TENG paired with Al, Cu, and PTFE, respectively. c–e) *I_sc_*, *V_oc_*, and *Q_c_* of the EVA@PPy‐based TENG at 1 Hz frequency. f) Voltage and current versus external load resistance. (g) Peak power density versus external load resistance. h) Long‐term stability test of the TENG over 10000 contact‐separation cycles.

The flexible conductive sponge can serve as both the triboelectric layer and electrode in a TENG.^[^ ^35^ ^]^ In this work, a single‐electrode TENG is fabricated using the prepared EVA@PPy sponge with a dimension of 38 mm × 38 mm × 7 mm (Figure S1a, Supporting Information). The effect of the EVA@PPy sponge thickness on the TENG's output performance is investigated, and the results in Figure S8 (Supporting Information) reveal that the TENG with the EVA@PPy sponge thickness of 6.5 mm shows the optimal output performance. To assess the electronegativity of the EVA@PPy sponge, the output performance of the EVA@PPy‐based TENG is tested by pairing it with materials of different polarities: Al and Cu (low electron affinity) and PTFE (high electron affinity). As shown in **Figure** **4**b, the TENG exhibits negligible *V_oc_* signals (≈0 V) when paired with Al or Cu, but generates significant outputs exceeding 9 V when contacted with PTFE. This pronounced contrast demonstrates the electron‐donating nature of the EVA@PPy sponge. In this case, the PTFE is selected to pair with the EVA@PPy‐based TENG to elucidate the working principle of the TENG.

The TENG operates based on the coupling effect of contact electrification and electrostatic induction.^[^ ^44^, ^45^, ^46^ ^]^ In Figure 4a, as the EVA@PPy‐based TENG contacts with the PTFE film, electrons on the EVA@PPy sponge surface transfer to the PTFE film surface, and equal charges of positive and negative are generated on their surfaces upon full contact (Figure 4a(i)). In the full contact state, no electron flows in the external circuit since the charges are balanced. Upon separating the PTFE from the EVA@PPy sponge, a potential difference is formed between the two materials, driving the electrons flow from ground to the EVA@PPy sponge and thus generating an electrical signal (Figure 4a(ii)). Electrostatic balance is reached when two materials completely separate (Figure 4a(iii)). When the PTFE film gradually approaches back to the EVA@PPy‐based TENG, the electrons flow from the EVA@PPy sponge to the ground, resulting in a reversed electrical signal (Figure 4a(iv)). Under contact‐separation cycles between the PTFE and the EVA@PPy‐based TENG at a frequency of 1 Hz, alternating electrical signals are generated. The prepared EVA@PPy‐based TENG achieved the *V_oc_* of 8 V, *I_sc_* of 0.9 µA, and *Q_sc_* of 12 nC, respectively, as shown in Figure 4c–e. In addition, the peak value of the power density is measured across various external resistances. As the resistance increases from 39 kΩ to 10 GΩ, the output voltage increases from 0.1 to 9.3 V, while the output current decreases from 1.2 to 0.3 µA (Figure 4f). And the power density reaches a maximum value of 7.8 mV m^−2^ at a resistance of 60 MΩ, see Figure 4g.

Figure S9 (Supporting Information) demonstrates that the *V_oc_* of the EVA@PPy‐based TENG exhibits a positive correlation with applied pressure. The *V_oc_* exhibits a steady increase from 0.25 to 1.25 V as the applied force increases from 0.9 to 24 N, which is attributed to the pressure‐enhanced contact area between the triboelectric layers. This linear response underscores the potential pressure‐sensing capability of the EVA@PPy‐based TENG. In addition, the EVA@PPy‐based TENG generates distinct electrical signals when tested against PTFE, PDMS, FEP, PMMA, PET, Cu, and Al. This indicates the ability of the EVA@PPy‐based TENG to detect various materials (Figure S10, Supporting Information). Moreover, the long‐term stability of the EVA@PPy‐based TENG is evaluated through 10000 contact‐separation cycles at 1 Hz frequency, as shown in Figure 4h. The consistent *V_oc_* confirms the reliable mechanical and electrical properties of the TENG, which highlights the robust adhesion of PPy to the EVA sponge during practical application.

### 2.4 Piezoresistive Effect of the EVA@PPy Sponge

**Figure 5:**
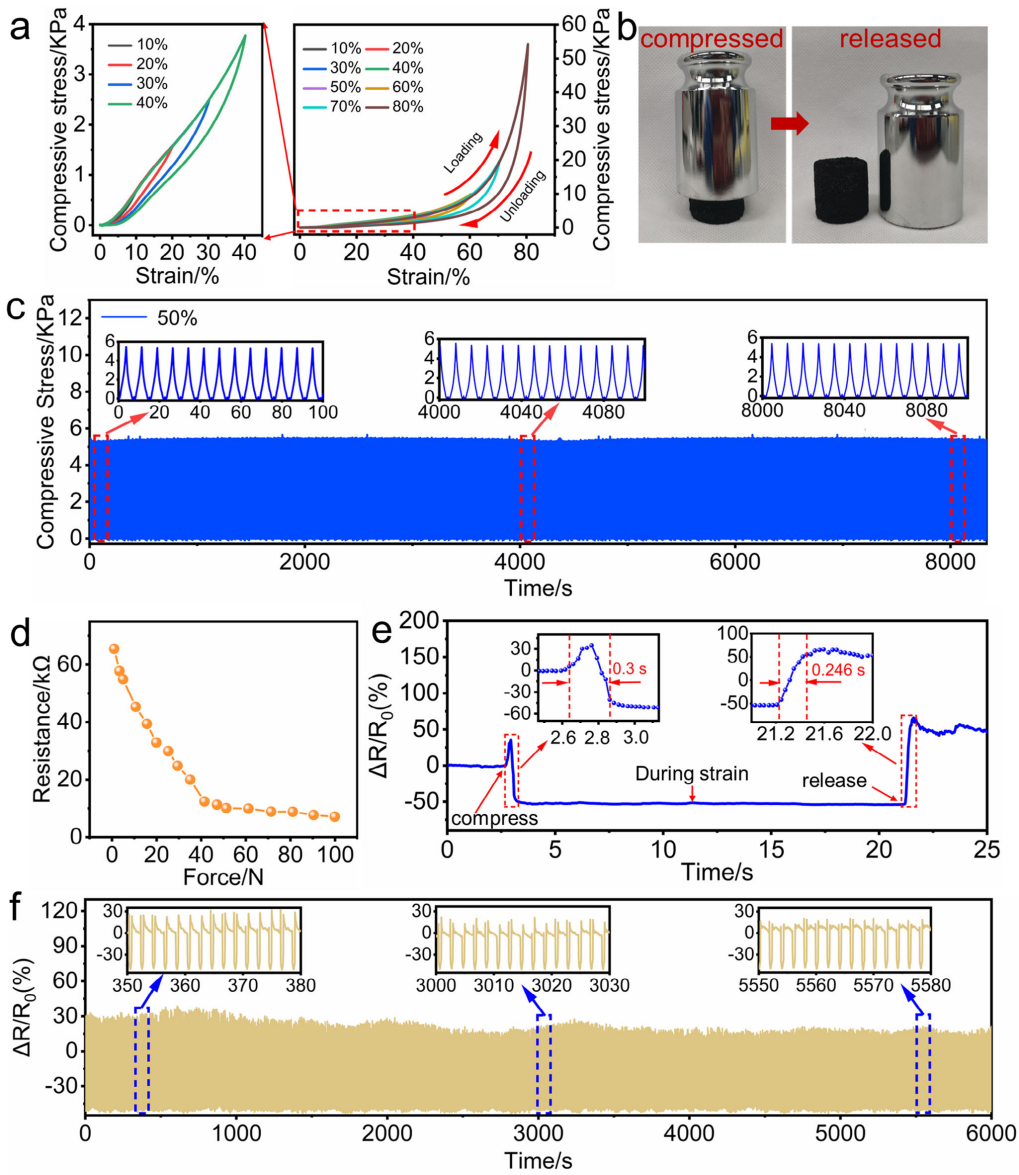
Piezoresistive effect of the EVA@PPy sponge. a) Stress–strain curves of the EVA@PPy sponge under varying compressive strains. b) Compressive and recovery properties of the EVA@PPy sponge. c) Robustness of the EVA@PPy sponge under 1000 cycles at 50% compressive strain. d) Force‐resistance curve of the EVA@PPy sponge. e) Response time of the EVA@PPy‐based piezoresistive sensor. f) Cycle response of the EVA@PPy‐based piezoresistive sensor up to 3000 cycles.

The introduction of abundant pores significantly lowers the material's density, which reduces the intrinsic stiffness of the structure due to the decreased solid content per unit volume. This lower stiffness is a fundamental prerequisite for high flexibility as it diminishes mechanical resistance to deformation.^[^ ^47^ ^]^ Under compressive stress, the pore walls undergo elastic bending, twisting, or buckling‐a highly reversible and low‐energy deformation mode‐which directly enables the material's compressibility and recoverability.^[^ ^18^ ^]^ The reversible compressibility of the prepared EVA@PPy sponge is evaluated through cyclic compression‐recovery tests. **Figure** **5**a presents the stress–strain curves of the EVA@PPy sponge during the cyclic tests at maximum strains of 10, 20, 30, 40, 50, 60, 70, and 80%. Furthermore, a low DH value of 4.3% is recorded during loading/unloading cycles at 80% compressive stress (Figure S11, Supporting Information), indicating its low hysteresis and rapid recovery capability.^[^ ^48^, ^49^ ^]^ Figure 5b displays the deformation and recoverability of the EVA@PPy sponge induced by mechanical motion. The results demonstrate the remarkable elasticity and reversible compressibility of the EVA@PPy sponge. The results also illustrate the low‐pressure hysteresis effect of the EVA@PPy sponge, which enables its high‐frequency response to external mechanical stimulation.^[^ ^50^ ^]^ Additionally, the EVA@PPy sponge shows superior compressive fatigue resistance, as evidenced by its ability to maintain nearly the same stress value after 1000 compression cycles at 50% strain (Figure 5c). These ensure its reliable mechanical properties for practical applications.

Figure 5d presents a significant decrease in the resistance of the EVA@PPy sponge from 66 to 7 kΩ as the applied force increases from 0 to 100 N, indicating the potential piezoresistive effect of the EVA@PPy sponge. To further assess the piezoresistive effect, the evolution in the relative resistance (*ΔR/R_0_*) value of the EVA@PPy‐based piezoresistive sensor during the compression‐recovery process is investigated. As seen in Figure 5d, the *ΔR/R_0_* initially increases from 0 to 30% followed by a decrease from 30 to −50% during compression. The *ΔR/R_0_* remains constant at −50% under the full compressed state. Upon release of the external pressure, the *ΔR/R_0_* increases to 50% before returning to the original value. Response time is a crucial parameter for evaluating the piezoresistive performance of sensors.^[^ ^51^ ^]^ As depicted in the inset of Figure 5e, the EVA@PPy‐based piezoresistive sensor exhibits a rapid response time of 300 ms under a pressure of 20 N. Furthermore, the *ΔR/R_0_* value remains stable after 3000 compression cycles under a pressure of 20 N (Figure 5f), which confirms the stable electromechanical performance of the EVA@PPy‐based piezoresistive sensor. In addition, the prepared piezoresistive sensors exhibit signal fluctuations over cycling. This is attributed to fatigue in the material's surface microstructure induced by repeated contact‐separation processes, which in turn causes minor variations in the effective contact area or contact efficiency during each cycle.

Moreover, the sensors fabricated using the EVA@PPy sponge exhibit high sensitivity, as shown in Figure S12 (Supporting Information). Compared with the sensing performance of the previously reported porous sensors, this sensor outperforms most of those listed in Table S1 (Supporting Information).

### 2.5 Applications of the EVA@PPy ‐Based Sensors in Human Motion Monitoring, Tactile Sensing, and Human Rehabilitation Training Recording

**Figure 6:**
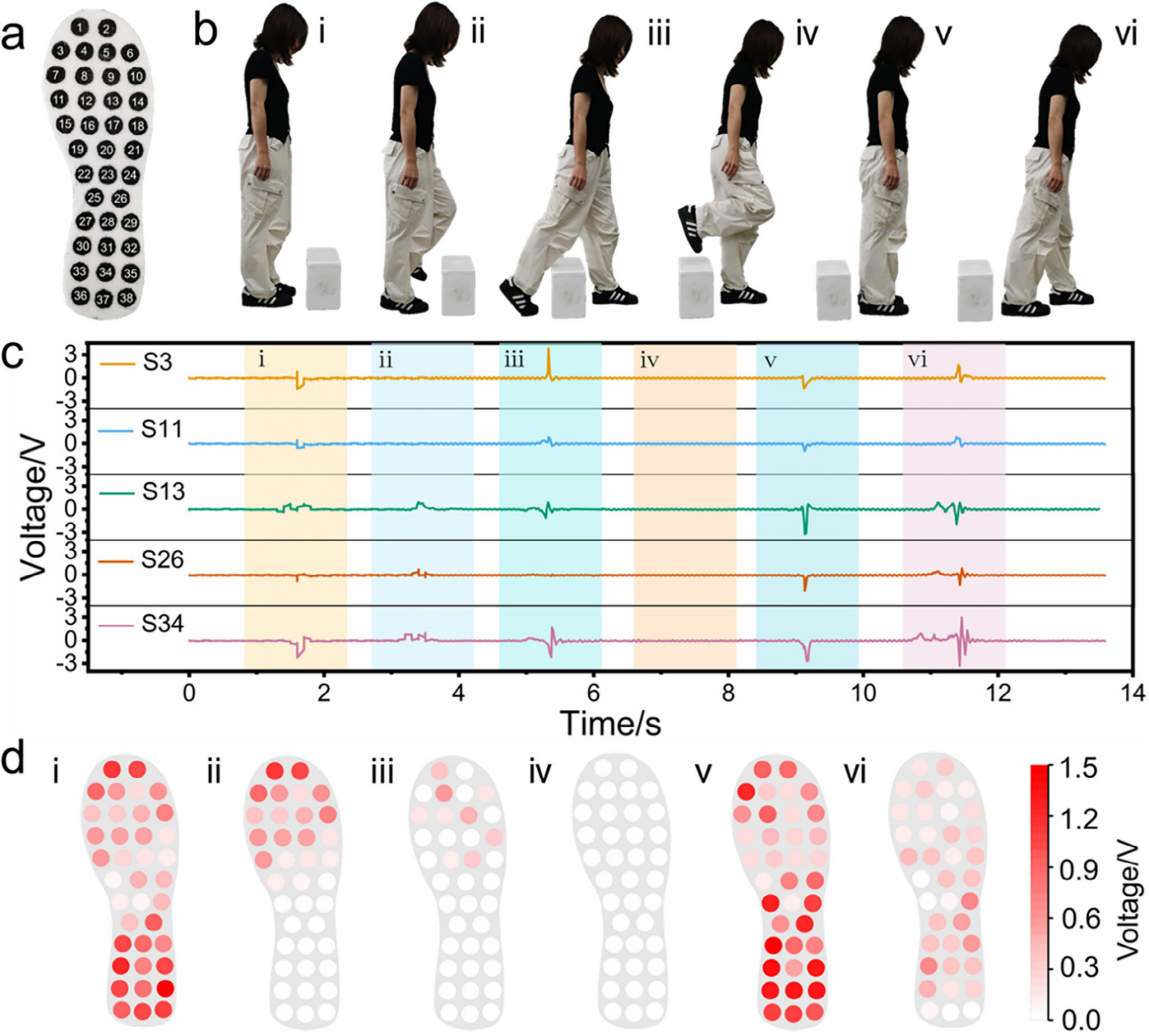
The EVA@PPy sponge was employed to fabricate a smart insole to monitor human plantar pressure. a) Photographs of the fabricated smart insole based on the EVA@PPy sponge. b) Demonstration of a volunteer performing an obstacle‐crossing motion. c) *V_oc_* signals from five representative sensors (S3, S11, S13, S26, and S34) on the smart insole during the obstacle‐crossing motion. d) Plantar pressure mapping images during the obstacle‐crossing motion, increased color saturation corresponds to a greater applied force.

The self‐powered sensors based on the triboelectric effect are capable of generating apparent *V_oc_* signals without an external power supply.^[^ ^52^, ^53^, ^54^ ^]^ Consequently, the EVA@PPy‐based TENG holds significant promise as self‐powered sensors for monitoring human motion through mechanical motion‐induced electrical signals. In this work, a smart insole based on the self‐powered EVA@PPy‐based sensor array is developed to monitor plantar pressure. In contrast to the reported smart insoles with embedded sensors,^[^ ^55^, ^56^ ^]^ the EVA sponge is employed as the fundamental material for fabricating the smart insole. By precisely controlling the vapor phase polymerization of PPy, an integrated sensor array is directly fabricated on the insole. As shown in **Figure** **6**a, the 38 sensors with a diameter (*d*) of 18 mm are arranged in a non‐matrix format at key pressure points, including the toe, sole, arch, and heel regions.^[^ ^57^ ^]^ The smart insole enables real‐time plantar activity detection through monitoring the *V_oc_* signals. (Video S2, Supporting Information). Additionally, each sensor is capable of independently tracking dynamic changes in plantar pressure without producing any mutual crosstalk in the array. This is verified by pressing a single sensor (S17) and simultaneously monitoring it along with three adjacent sensors (S9, S12, and S13). As shown in Figure S13 (Supporting Information), clear triboelecric signals were only generated from S17, while S9, S12, and S13 remained unresponsive.

The performance of the prepared smart insole is evaluated by detecting an obstacle‐crossing task. Six crucial moments, including (i) standing, (ii) lifting the foot, (iii) getting off the ground, (iv) crossing the obstacle, (v) landing, and (vi) leaving, are captured using a digital camera (Figure 6b). Figure 6c presents the *V_oc_* signal variation of the representative five channels (S3, S11, S13, S26, S34) in the sensor array of the right foot as the participant executes the task. Valleys and peaks on the curves corresponding to the six moments indicate pressure release and application on the insole, respectively. 2D pressure mappings of the six moments are acquired from the entire sensor array (Figure 6d), which displays the plantar pressure distribution and reflects the change of the participant's body gravity center. As the participant prepares to cross the obstacle, the right plantar pressure points gradually decrease, and the gravity center shifts forward (Figure 6d(i–iii)). The pressure on the right foot is zero when the foot is completely lifted (Figure 6d(iv)). Upon landing, plantar pressure distribution becomes even and reaches maximum owing to the entire body weight (Figure 6d(v)). The following leaving behaviour causes the decrease of plantar pressure (Figure 6d(vi)). These results demonstrate that the smart insole can dynamically map plantar pressure, aiding in exercise state assessment.

**Figure 7:**
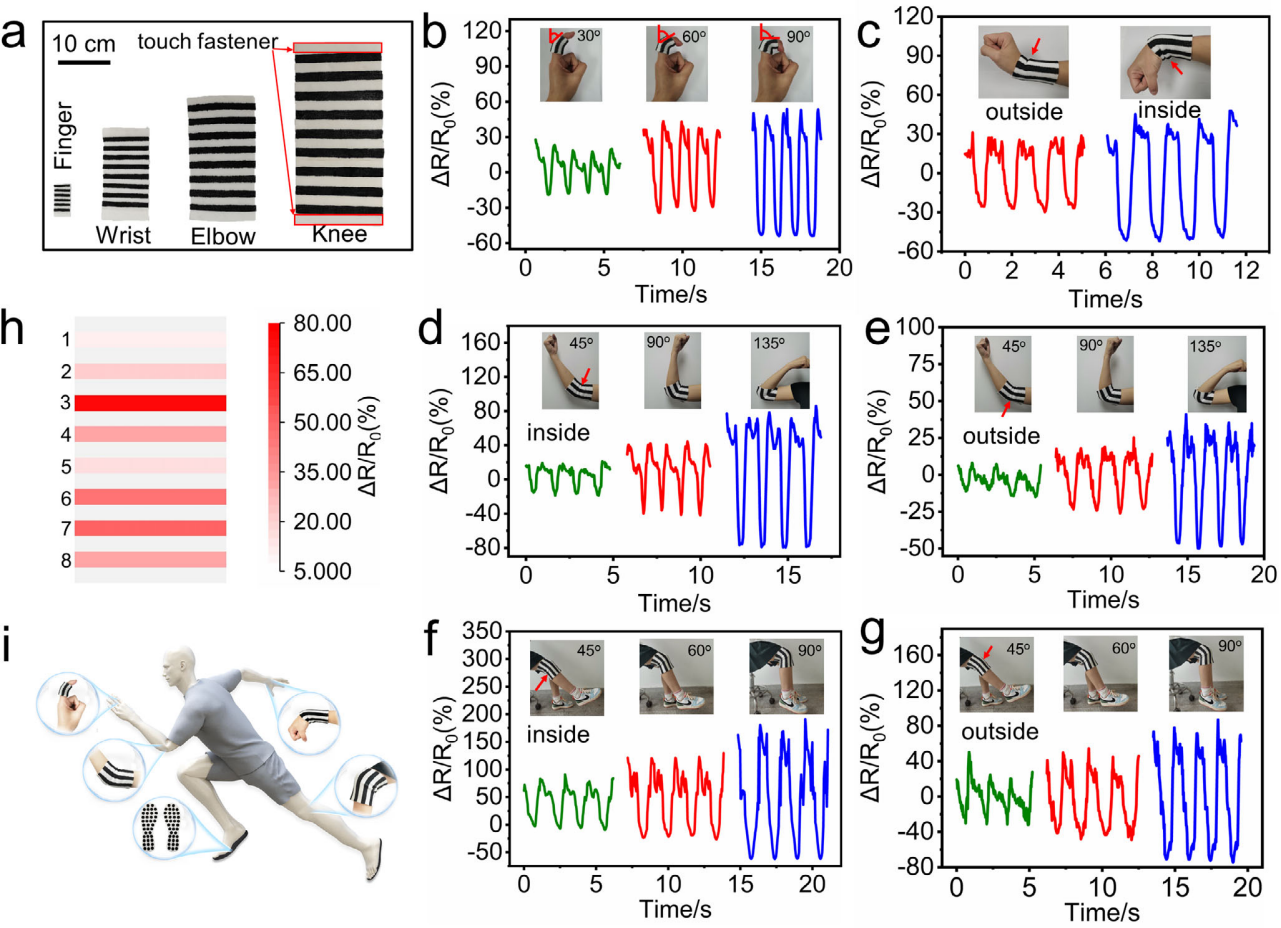
The application of the EVA@PPy‐based piezoresistive sensor in multi‐joint movement detection. a) Photographs of fabricated joint bands for finger, wrist, elbow, and knee. b) Relative resistance changes during finger bending angle ranging from 0° to 30°, 60°, and 90°, respectively. c) Relative resistance changes of inside and outside of the wrist during bending up and down. d, e) Relative resistance changes of inside and outside of the elbow during the bending angle ranging from 0° to 45°, 90°, and 135°. f, g) Relative resistance changes of the inside and outside of the knee during the bending angle ranging from 0° to 45°, 90°, and 135°. h) Motion mapping image of elbow segments at a 135° bending. i) An illustration of body movement detection based on the EVA@PPy‐based sensor.

In addition, various smart joint bands are designed and fabricated by piezoresistive sensors based on the prepared EVA@PPy sponge to monitor human joint motions, including finger bending, wrist bending, elbow bending, and knee bending, as shown in **Figure** **7**a. The two terminals of the smart bands are connected through a touch fastener when worn on joints. To overcome the inherent spatial resolution limitations in conventional single‐sensor configurations, we implement a sensor array design for each joint band. In Figure 7b, as the participant's finger bends from 0° to 30°, 60°, and 90°, the *ΔR*/*R_0_* peak values increase to −16%, −32%, and −53%, respectively. As shown in Figure 7c–g, the bands can also monitor bending motions of the wrist, elbow, and knee, showing similar trends in the variations of the *ΔR*/*R_0_* peak value. Moreover, Figure 7d–g demonstrates that the bands can simultaneously detect both medial and lateral displacement during flexion‐extension cycles of both elbow and knee, indicating the sensor array enables synchronous monitoring of movement at multiple locations on individual joints. Accordingly, the spatial distribution of motion across various segments is to be visualized exhibition (Figures 7h and S14, Supporting Information). These demonstrate that the smart bands can effectively detect bending activities of various joints, and the bending degree of the joints can be determined through signal peak value analysis. In combination with the plantar pressure detection, comprehensive human activities can be effectively monitored, as illustrated in Figure 7i.

**Figure 8:**
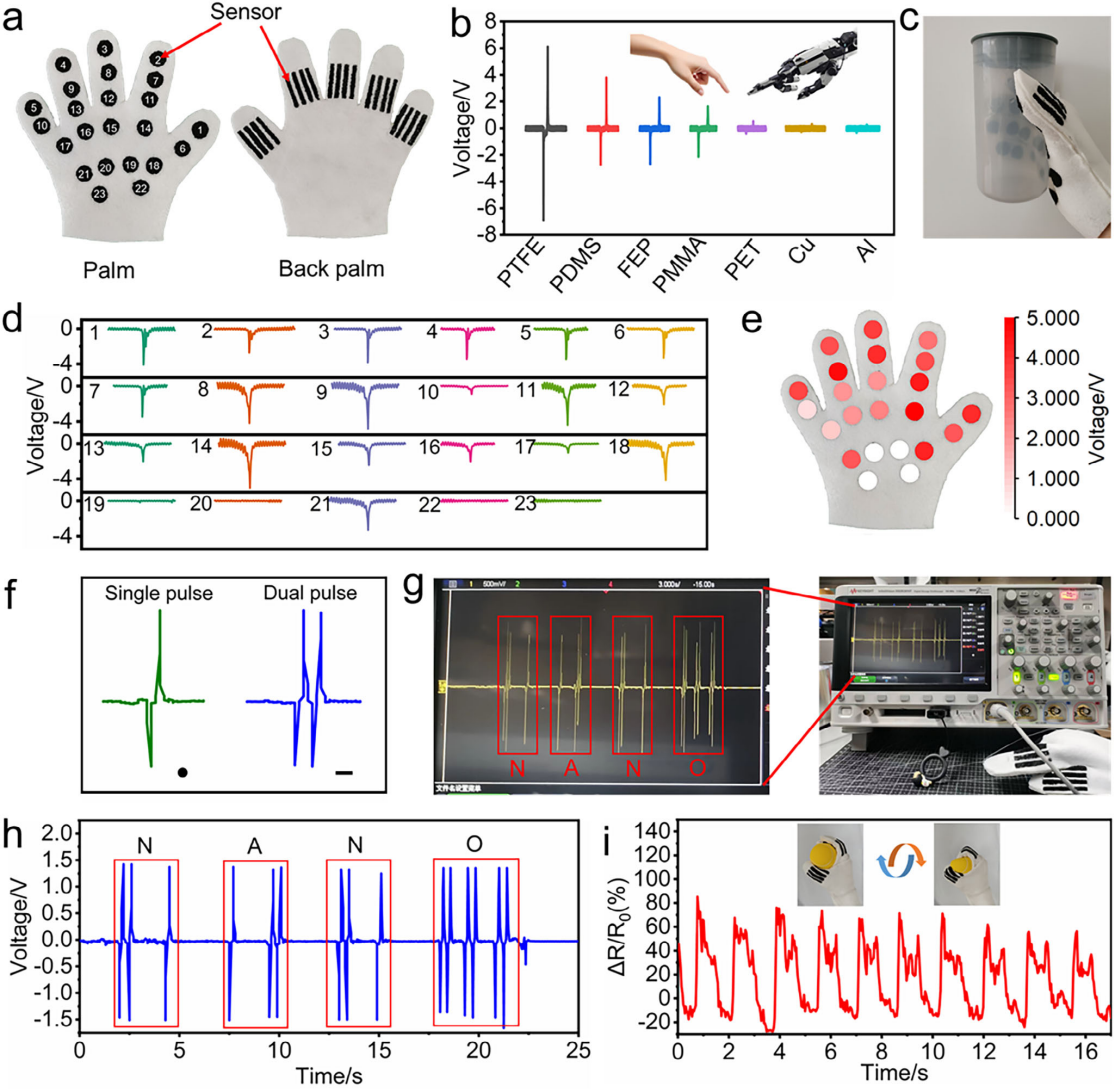
Demonstration of the smart glove with the EVA@PPy‐based sensor array for object recognition, palm pressure distribution mapping, Morse code communication, and human rehabilitation training recording. a) Photographs of the fabricated smart glove (palm and dorsal views). b) *V_oc_* levels detected by the smart glove sensors when contacting various materials. c) Optical photograph of a gripping cup. d) *V_oc_* signals of the sensors at various positions when holding the cup. e) Palm pressure mapping images. f) Encode the output signal into the input signal of Morse code. g,h) The information simulation and identification of the string “NANO” enabled by the glove sensor 2. i) The sensor detects signal changes during rehabilitation training.

Furthermore, a smart glove integrated with sensor arrays on both the palm and dorsal side is designed and fabricated (**Figure** **8**a), which is capable of object recognition, palm pressure distribution mapping, Morse code communication, and human rehabilitation training recording. The triboelectric sensors on the palmar side of the smart glove generated distinct output signals when interacting with diverse materials, whether worn by human or robot hands. As illustrated in Figure 8b, the triboelectric signals are progressively weak upon contact with PTFE, PDMS, FEP, PMMA, PET, Cu, and Al, which is consistent with the electronegativity difference between these materials and the EVA@PPy sponge. In addition, the controllable vapor‐phase polymerized PPy enables exceptional sensor array uniformity, allowing precise spatial mapping of palmar pressure during object grasping (Figure 8c–e). The triboelectric interface facilitates Morse code transmission through encoding short (“•”) and long (“─”) signals corresponding to single and dual pulses, respectively (Figures 8f and S15, Supporting Information). This capability is demonstrated by successfully transmitting the “NANO” sequence (Figure 8g,h). Moreover, piezoresistive sensors on the glove's dorsal side enable real‐time finger kinematic tracking during rehabilitation ball grasping exercises (Figure 8i), highlighting the system's potential application for rehabilitation therapy monitoring.

Besides the smart insole and glove, the EVA@PPy‐based sensor array can also be attached to the robot surface to function as artificial skin, as shown in Figure S16a,b (Supporting Information). Upon touch, it generates a distinct negative electrical signal, which switches to positive upon release, as demonstrated in Figure S16c,d (Supporting Information). This signal response enables the sensor array to accurately identify the touch location on the robot, as shown in Figure S16e,f (Supporting Information).

Table S2 (Supporting Information) compares the prepared EVA@PPy‐based sensors with previously reported flexible porous sensors. As seen, the as‐fabricated sensor shows outstanding comprehensive performance, including self‐recoverability and dual sensing modes. This underscores the fabricated sensor's significant potential for developing next‐generation advanced wearable devices, human‐machine interfaces, artificial intelligence, and robotics.

## 3 Conclusion

In this work, a self‐recoverable flexible porous sensor with diverse designability of electrodes was fabricated, which is capable of addressing the performance degradation, adapting to various human body contours, and detecting comprehensive human motion. A conductive shape memory EVA@PPy sponge was developed to fabricate the porous electrode. The sponge was prepared by creating pores in EVA using a salt sacrificial template method, followed by vapor phase polymerization of PPy. This fabrication approach employs two methods, stencil‐based patterning and freehand drawing, to customize electrode shapes, which ensures sensor compatibility with diverse body contours. In addition, benefiting from the shape memory effect of the EVA@PPy sponge, the sensors displayed enhanced performance and structural robustness as they can recover to their original structure under thermal stimulus. Furthermore, the sensors based on the EVA@PPy‐based sponge exhibited both triboelectric and piezoresistive effects. Consequently, a self‐powered smart insole based on the sensors was fabricated to detect dynamic human plantar pressure, presenting an excellent sensing sensitivity for pressure stimuli. Various smart joint bands based on the EVA@PPy‐based piezoresistive sensors were also manufactured to track joint movements of human beings. With the help of an integrated sensor array, the joint bands enabled simultaneous multi‐position detection on individual joints, effectively addressing the challenge of detecting concurrent movements at a single joint. Moreover, an intelligent glove was fabricated to recognize different objects, track palm pressure distribution, transmit Morse code, and monitor rehabilitation training. These results certify that the self‐recovering flexible porous sensor is reliable, stable, and promising for wearable devices, which will inspire the development of next‐generation wearable sensors, human‐machine interfaces, artificial intelligence, and robotics.

## 4 Experimental Section

### Materials

Ethylene‐vinyl acetate (EVA, 20% VA) and Pyrrole (Py) from Shanghai Aladdin Biochemical Technology Co., Ltd were used. Cyclohexane and Ferric chloride hexahydrate (FeCl_3_·6H_2_O) were obtained from Shanghai Macklin Biochemical Technology Co., Ltd. Sodium chloride (NaCl) was applied by Xuetian Co., Ltd. All chemicals were used without further purification.

### Fabrication of EVA Sponge

EVA, a well‐documented SMP, was employed to fabricate the sponge using a sacrificial template method.^[^ ^58^ ^]^ The EVA pellets were dissolved in cyclohexane at 65 °C under magnetic stirring for 1 h to obtain a homogeneous solution. The mass ratio of EVA: Cyclohexane was 1:5.^[^ ^33^ ^]^ Subsequently, a certain amount of NaCl particles (≈ 500 µm) was used as the sacrificial template and uniformly mixed with the solution. The mass ratio of EVA: NaCl was set at 1:22 to create pores in the sponge as much as possible. The NaCl particles could not be mixed as the mass ratio of EVA: NaCl was higher than this value. The mixture was poured into a quartz mold and then moved to a fuming cupboard. After evaporation of the cyclohexane for 24 h, the solidified mixture was demolded from the mold and immersed in deionized water until the NaCl particles were completely removed. Afterward, the rest samples were dried under ambient conditions to obtain the EVA sponges.

### Fabrication of EVA@PPy Sponge

Since PPy was an electroconductive polymer (ECP) with high conductivity and robust mechanical properties,^[^ ^59^, ^60^, ^61^ ^]^ it was integrated with the prepared sponge to endow the EVA sponge with conductivity. The as‐prepared EVA sponge was immersed in a 0.1 m FeCl_3_ solution. Then the FeCl_3_‐coated sponge was transferred to a culture dish and placed in a vacuum desiccator containing a separate culture dish with Py solution. Vapor phase polymerization of PPy was carried out under a pressure of −0.1 mPa. By controlling the reaction time (15–90 min), various PPy‐coated sponges (EVA@PPy) were acquired. Subsequently, the EVA@PPy sponges were rinsed with deionized water to eliminate potential unreacted reagents, followed by drying under ambient conditions.

### Fabrication of the EVA@PPy sponge‐Based Sensors

Triboelectric sensors based on a single‐electrode triboelectric nanogenerator (TENG) were fabricated as illustrated in Figure S1a (Supporting Information). Piezoresistive sensors were fabricated by attaching two wires to the EVA@PPy sponge using two flexible copper tape sheets, see Figure S1b (Supporting Information).

### Characterization and Measurement

The porous structure of the as‐prepared EVA@PPy sponge was characterized by a scanning electron microscope (SEM) (Quanta TM 250 FEG, USA). The open‐circuit voltage (*V_oc_*), short‐circuit current (*I_sc_*), and transferred charge (*Q_c_*) were measured using an oscilloscope (LeCroy 44MXI Wave Runner, USA), a low noise current amplifier (Stanford Research System SR570, USA), and an electrometer (Keithley Model 6514, USA), respectively. The electrical conductivity was recorded using a source meter (Keithley 2400, USA) and a multimeter. The compressive stress was evaluated using a universal material testing machine (CMT4204, China). A source meter (Keithley 2450, USA) was used to record the resistance change of the sensor under the applied voltage. The relative resistance change was calculated by *ΔR*/*R_0_* (%) = (*R*‐*R_0_*)/*R_0_* × 100%, in which *R_0_* refers to the initial resistance and *R* means the resistance after deformation.

The shape‐memory effect of the as‐prepared EVA and EVA@PPy sponges was assessed based on angle variation and compressive deformation. The test specimens were placed in a drying oven at 65 °C (glass transition temperature, *T_g_*) for 5 min, and then compressed together with an M‐shape mold for an additional 5 min. Afterward, the compressed specimens were cooled to room temperature, and subsequently placed back into the drying oven at 65 °C to observe their recoverability.

## Conflict of Interest

The authors declare no conflict of interest.

## Supporting information



Supporting Information

Supplemental Movie 1

Supplemental Movie 2

## Data Availability

The data that support the findings of this study are available from the corresponding author upon reasonable request.
