# Supplementary material for: Thermo‐Responsive Self‐Recoverable Porous Sensors with Writable Electrodes: Advancing Wearable Motion Detection
Source: Adv Sci (Weinh). 2025 Dec 16;13(12):e17254. doi: 10.1002/advs.202517254 (PMC12948277; doi:10.1002/advs.202517254)
Supplement: Supplementary file 1 — Supporting Information [file ADVS-13-e17254-s003.docx]

**Thermo-Responsive Self-Recoverable Porous Sensors with Writable Electrodes: Advancing Wearable Motion Detection**

*Ying Gao^1,2^, Xingyi Dai^3^,* *Y**uanyuan Zhang^2^, Guohao Fang^5^,* *Gui Li^1^,* *Yu Zheng^1^, Long-Biao Huang^3,4^*, Biqin Dong^2^**

^1^Dr. Y. Gao, Dr. G Li, Dr. Y Zheng

School of Environment and Civil Engineering, Guangdong Provincial Key Laboratory of Intelligent Disaster Prevention and Emergency Technologies for Urban Lifeline Engineering, Dongguan University of Technology, Dongguan, 523808, P. R. China

^2^Dr. Y. Gao, Dr. Y. Zhang Dr. B. Dong

College of Civil and Transportation Engineering, Guangdong Province Key Laboratory of Durability for Marine Civil Engineering, The Key Laboratory on Durability of Civil Engineering in Shenzhen

Shenzhen University, Shenzhen 518060, P. R. China

*Corresponding Author, E-mail: [incise@szu.edu.cn](mailto:incise@szu.edu.cn)

^3^Dr. X. Dai, Dr. L.-B. Huang

Key Laboratory of Optoelectronic Devices and Systems of Ministry of Education and Guangdong Province, College of Physics and Optoelectronic Engineering

Shenzhen University, Shenzhen 518060, P. R. China

*Corresponding Author, E-mail: [huanglb@szu.edu.cn](mailto:huanglb@szu.edu.cn)

^4^Dr. L.-B. Huang

National Key Laboratory of Green and Long-Life Road Engineering in Extreme Environment

Shenzhen University, Shenzhen 518060, P. R. China

*Corresponding Author, E-mail: [huanglb@szu.edu.cn](mailto:huanglb@szu.edu.cn)

^5^Dr. G. Fang

Institute for Advanced Study

Shenzhen University, Shenzhen 518060, P. R. China


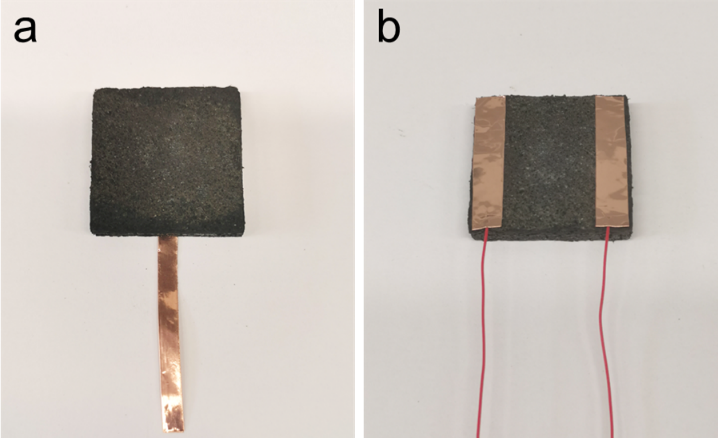


**Figure S1.** Photographs of the a) EVA@PPy-based triboelectric sensor and b) EVA@PPy-based piezoresistive sensor.

The moisture permeability of the sensor is characterised at 25 ^o^C and 38 ^o^C, as shown in the figure below. The moisture permeability of the sensor prepared is 71.37 gm^-2^h^-1^ at 25 ^o^C and 20.84 gm^-2^h^-1^ at 38 ^o^C, as shown in **Figure S2**, laying the foundation for long-term wear comfort. Besides, a temperature rise is better for the moisture permeability improvement of the sensor, which is related to the evaporation rate of water vapour ^[1, 2]^.





**Figure S2.** The moisture permeability of the prepared EVA@PPy sponge at 25 ^o^C and 38 ^o^C.





**Figure S3.** FTIR spectra of the EVA sponge and EVA@PPy sponge.

The width of the line is determined by the line openings in the stencil. Thus, the width of the line can be furtherly reduced to 0.5 mm, as shown in **Figure S4a**.

Moreover, the measured resistance of prepared lines with different widths is presented in **Figure S4b**. All straight lines show a correlation factor of higher than 0.9, indicating excellent uniformity of the prepared lines at various line widths.

To further evaluate uniformity, various shapes with different sizes are also fabricated using the stencil method, as displayed in **Figure S4c**. When the side length of the square is 0.2 cm, the shape becomes unrecognisable. Thus, the minimum feasible side length for a square is 0.2 cm. Similarly, the limiting side lengths for the pentacle and triangle are 0. 3 and 0.2 cm, respectively.


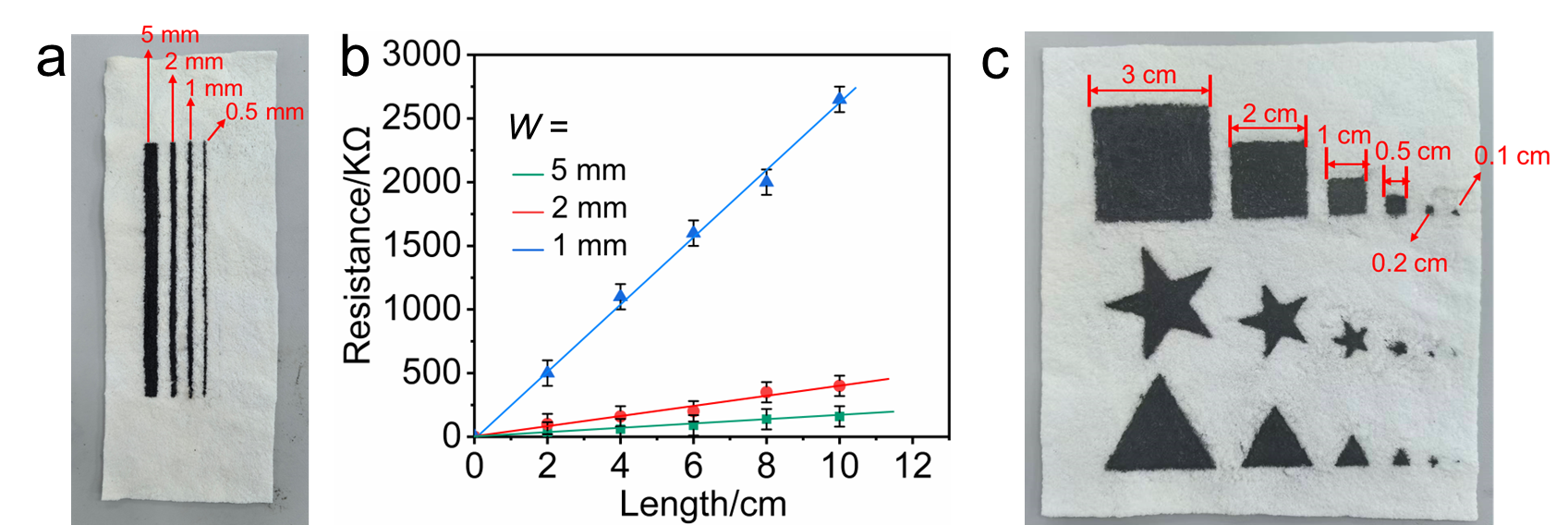


**Figure S4.** a) Photograph of straight lines with various widths, including 5, 2, 1, and 0.5 mm. b) Measured the resistance of the prepared line with widths of 5, 2, and 1 mm. c) Photograph of patterned structures with different geometries and dimensions.


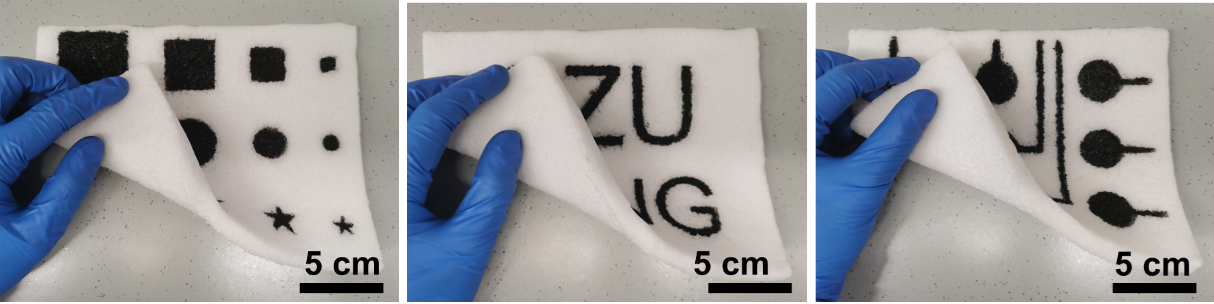


**Figure S5.** Photographs of selectively formed PPy patterns on EVA sponge surfaces.


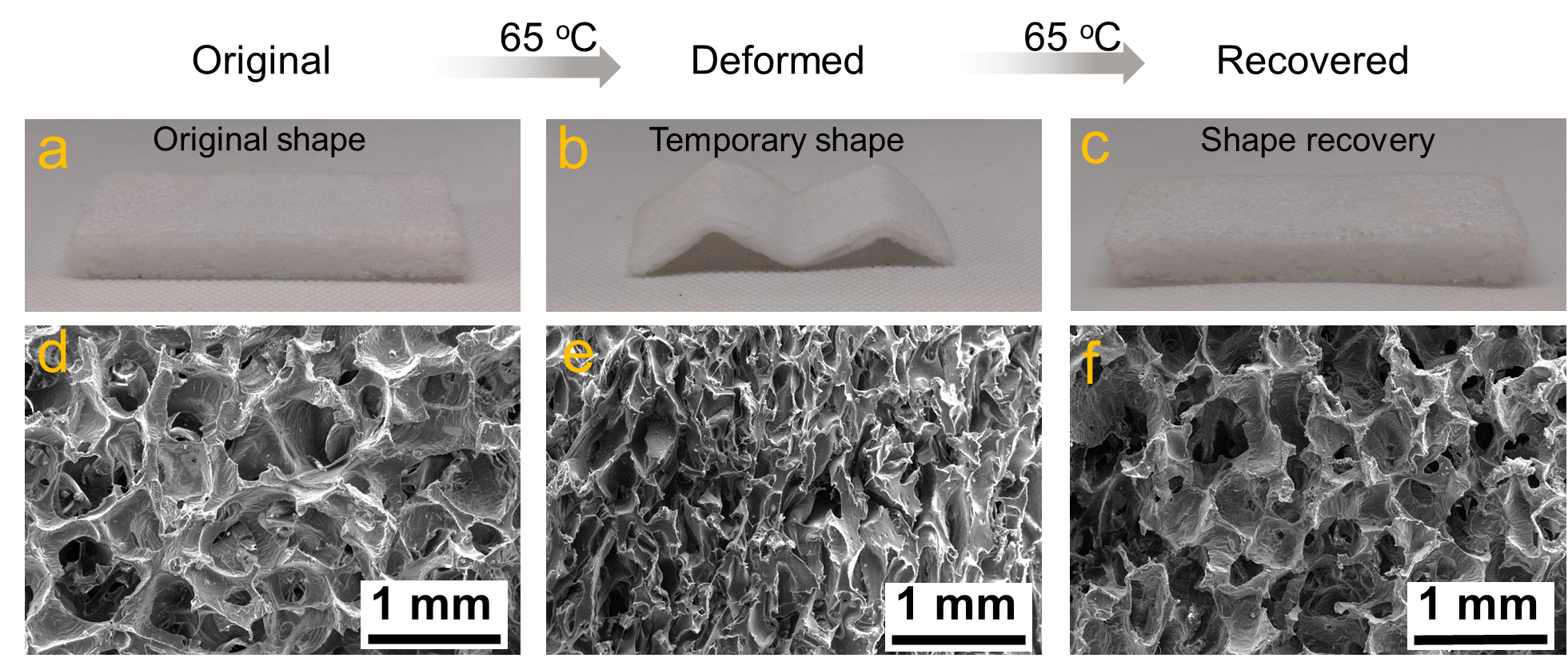


**Figure S6.** Morphological characterization during deformation-recovery: a-c) macroscopic photographs and d-f) SEM micrographs of EVA sponge.





**Figure S7.** *V_oc_* of the EVA@PPy-based TENG in original, deformed, and recovered states.


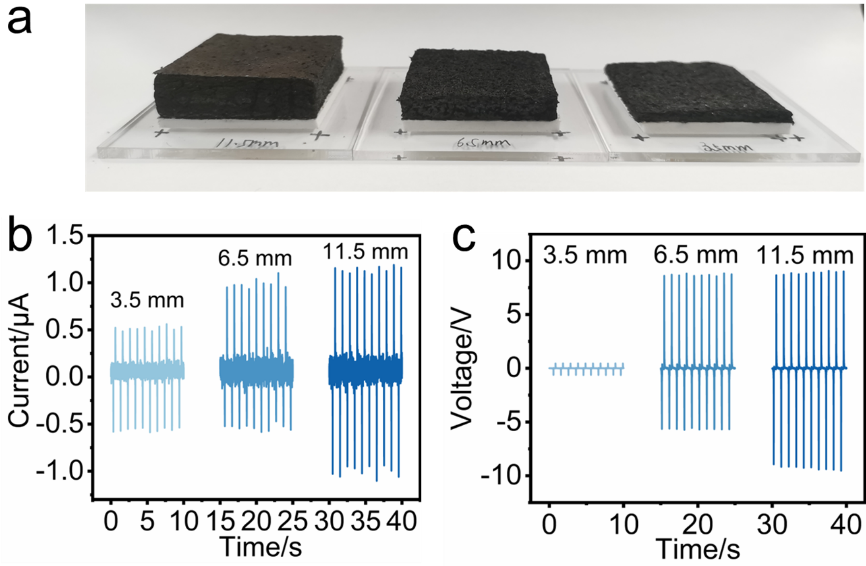


**Figure S8.** The morphology of the EVA@PPy sponges with various thicknesses and the output of the EVA@PPy-based TENGs. a) Photographs of the EVA@PPy sponge with thicknesses of 11.5 mm, 6.5 mm, and 3.5 mm (from left to right). b, c) *I_sc_* and *V_oc_* of the corresponding TENG.





**Figure S9.** *V_oc_* response of the EVA@PPy-based TENG under applied pressure ranging from 0.9 to 24 N.





**Figure S10.** *V_oc_* response of the EVA@PPy-based TENG to various contact materials.

The degree of hysteresis (DH) is defined as follows:

DH=(*A_loading_*-*A_unloading_*)/*A_loading_*×100%

Where *A_loading_* and *A_unloading_* are the areas of loading and unloading response curves, respectively. Small DH indicates lower hysteresis of the sensor. ^[3, 4]^ The *A_loading_* and *A_unloading_* are displayed in **Figure S11**, the calculated DH value of the prepared EVA@PPy sponge is 4.3% during the loading/unloading process at an 80% comprehensive stress, indicating low hysteresis and a rapid recovery to capability of the EVA@PPy-based sensor.


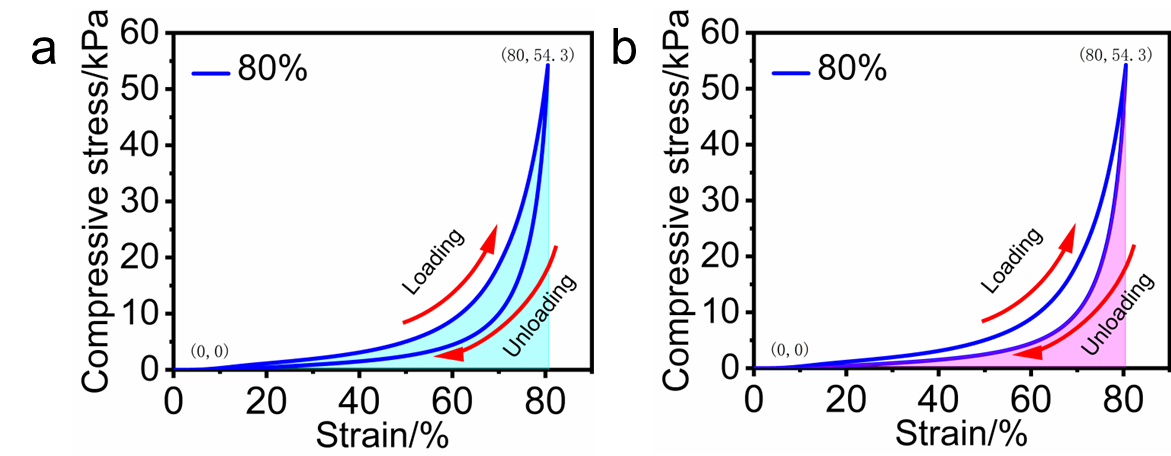


**Figure S11.** The areas of loading a) and unloading b) response curves.

The relative voltage change-pressure relationship of the sensors is systematically investigated. The force applied by the motor ranged from 1.65 N to 96.7 N. The contact area between the sample and the contact material was 3.7 cm × 3.7 cm. Thus, the corresponding pressure range applied to the sample was 1.21-70.635 kPa. The sensitivity (*S*) is defined as the slope of the curve S = (*δ*(*ΔV*/*V_0_*) / *δP*), where V_0_ is the magnitude of the corresponding output voltage at an external pressure of 1.21 kPa. The equation for the change in voltage is *ΔV* = *V* − *V_0_*, and *V* is the voltage in response to a specific pressure in the range of 1.21-70.635 kPa. *δP* refers to the change of pressure.

In addition, for a piezoresistive sensor, the calculation of sensitivity is based on the formula *S* = (*δ*(*ΔR* / *R_0_*) / *δP*), where *ΔR*/*R_0_* referes to the relative resistance change, and *δP* refers to the change of pressure. The sensitivity is defined as S1, S2, and S3 in the pressure range (0.73 ~ 7.3 kPa), (7.3 ~ 21.91 kPa), and (21.91 ~ 34.68 kPa).

The sensitivity of the triboelectric sensor is 0.0515 kPa^−1^ in the pressure range of 1.21 ~ 22.2 kPa, and 0.0189 kPa^−1^ in the pressure range of 22.2 ~ 51.5 kPa. The sensitivity of the piezoresistive sensor is -2.8875 kPa^−1^ in the pressure range of 0.73 ~ 7.3 kPa, -0.8219 kPa^−1^ in the pressure range of 7.3 ~ 21.91 kPa, and -0.4518 kPa^−1^ in the pressure range of 21.91 ~ 69.39 kPa. Compared with the sensing performance of the previously reported triboelectric porous sensors, this sensor outperforms most of those listed in **Table S1**.


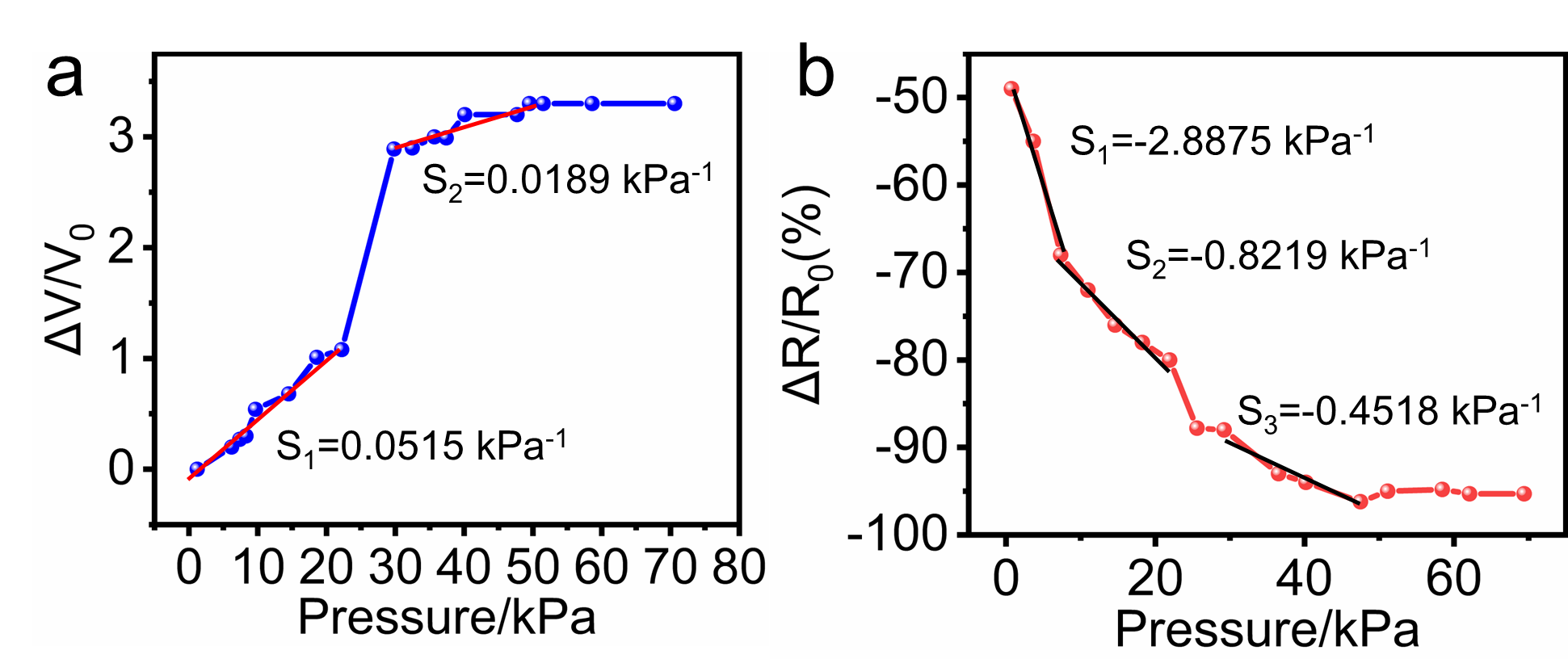


**Figure S12.** The sensitivity of the prepared sensors. a) Relative voltage change-pressure curves of the prepared tribelectric sensor. b) Relative resistance change-pressure curves of the prepared piezoresistive sensor.

To investigate the crosstalk in the array, the signals of four adjacent sensors (S9, S12, S13, and S17) in the smart insole are detected simultaneously when only S17 is pressed. As shown in **Figure S13**, a distinct signal was observed when pressing S17, while no signals were captured from S9, S12, and S13 channels, indicating there is no crosstalk in the array.


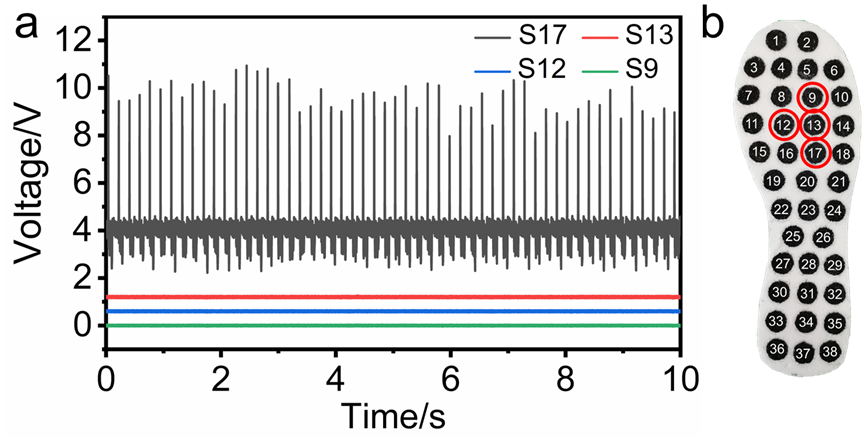


**Figure S13.** The crosstalk in the sensor array. a) Voltage response obtained from S9, S12, S13, and S17 when pressing S17. b) The displacement of the selected sensors.


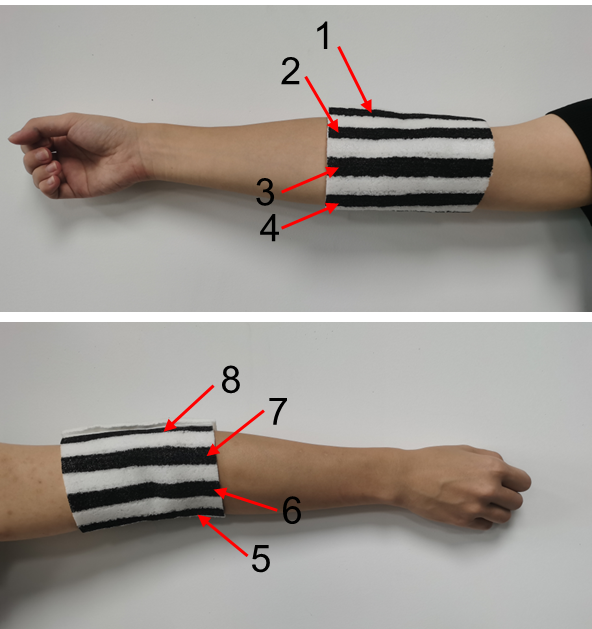


**Figure S14.** The position numbers of EVA@PPy piezoresistive sensors on the elbow band.


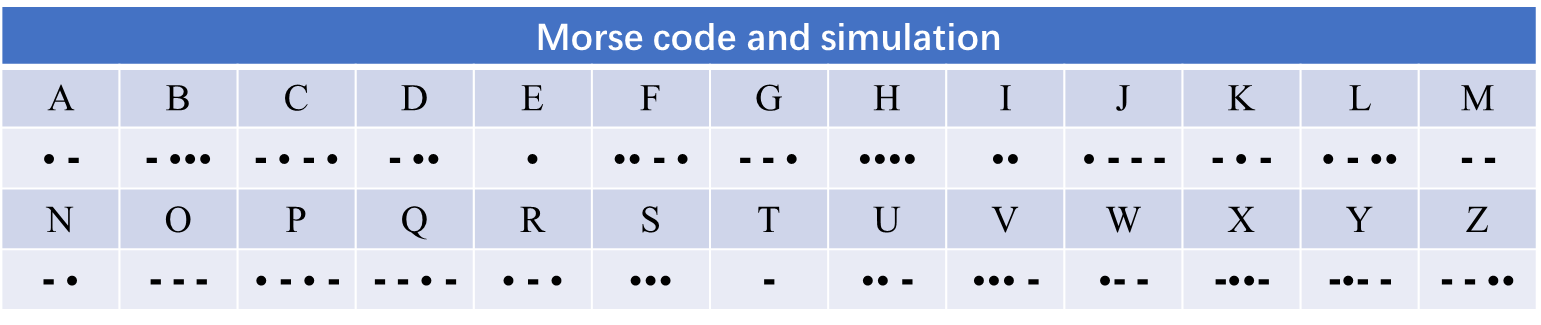


**Figure S15.** Part of Morse code.


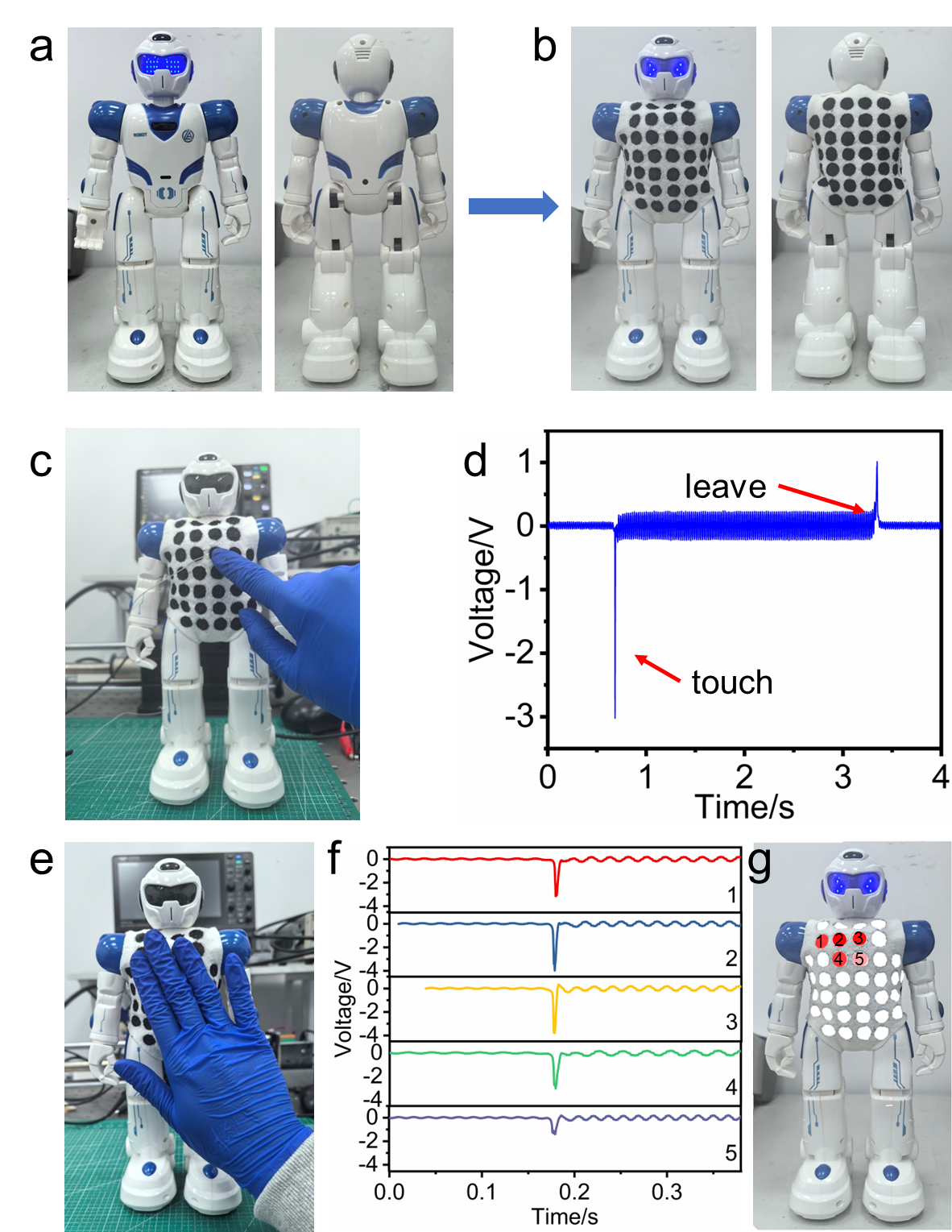


**Figure S16.** The application of the EVA@PPy-based sensor array in robot skin.the application of a) Schematics of the robot’s front and back views. b) Schematics of the robot’s front and back views with the skin attached. c) Photograph of a touch event on the robot. d) The corresponding electrical signals responded by the sensor array on the robot skin. e) Photograph of a hand touching the robot. f) *V_oc_* signals of the sensors at the touched positions. g) Mapping image of the touched position.

**Table S1**

Sensing performance of the main parameters between our work and recently reported porous sensors.

| References | Working mechanism | Detection range | Sensitivity |
| --- | --- | --- | --- |
| [5] | Triboelectric | 4.4 ~ 31.1 kPa | 0.017 V kPa^−1^ |
| [6] | Triboelectric | 0 ~ 50 kPa  50 ~ 150 kPa | 0.017 kPa^-1^  0.003 kPa^-1^ |
| [7] | Triboelectric | 0 ~ 40 kPa | 0.011 kPa^-1^ |
| [8] | Triboelectric | 2.5 ~ 25 kPa | 0.0188 V kPa^-1^ |
| [9] | Triboelectric | 0~5 kPa  5~50 kPa | 0.57 kPa^-1^  0.04 kPa^-1^ |
| [10] | Triboelectric | 0~150 kPa | 1.76 kPa^-1^  0.44 kPa^-1^  0.19 kPa^-1^ |
| [11] | Piezoresistive | 0 ~ 30 kPa  30 ~ 100 kPa | 0.014 kPa^-1^  0.009 kPa^-1^ |
| [12] | Piezoresistive | 0.001 ~ 1 kPa  1 ~ 10 kPa | 10.805 kPa^-1^  2.015 kPa^-1^ |
| [13] | Piezoresistive | 0 ~ 80 kPa | 0.309 kPa^-1^ |
| [14] | Piezoresistive | 0 ~ 260 kPa | 0.59 kPa^-1^ |
| [15] | Piezoresistive | 0~2 kPa  2~20 kPa | 17.2 kPa^-1^  0.012 kPa^-1^ |
| [16] | Piezoresistive | 0 ~ 10 kPa  10 ~ 30 kPa  30 ~ 200 kPa | 8.0033 kPa^-1^  0.5921 kPa^-1^  0.0695 kPa^-1^ |
| [17] | Piezocapacitive | 0 ~ 1000 Pa  100 kPa | 92.87 kPa^-1^  1.12 kPa^-1^ |
| [18] | Piezocapacitive | < 5 kPa | 0.759 kPa^-1^ |
| [19] | Piezocapacitive | 0 ~ 1 kPa  30 ~ 50 kPa | 3.13 kPa^-1^  0.43 kPa^-1^ |
| This work | Triboelectric- piezoresistive | 1.21 ~ 22.2 kPa  22.2 ~ 51.5 kPa | 0.0515 kPa^−1^  0.0189 kPa^−1^ |
|  |  | 0.73 ~ 7.3 kPa  7.3 ~ 21.91 kPa  21.91 ~ 47.48 kPa | -2.8875 kPa^−1^  -0.8219 kPa^−1^  -0.4518 kPa^−1^ |

**Table S2.**

Comparison of the flexible porous sensor in this work with the previously reported flexible porous sensor.

| Materials | Self-recoverability | Conductivity | Sensing mode | | Ref. |
| --- | --- | --- | --- | --- | --- |
|  |  |  | Triboelectric mode | Piezoresistive mode |  |
| CNT_S_/PDMS | No | Yes | Yes | Yes | [20] |
| CI–Ecoflex | No | No | Yes | No | [21] |
| PDPU | No | No | Yes | No | [22] |
| PU/PANI | No | Yes | Yes | No | [23] |
| CNT_S_/PDMS | No | Yes | No | Yes | [24] |
| CNT@PU/silicone | No | Yes | Yes | No | [25] |
| carbon | No | Yes | Yes | Yes | [26] |
| PDMS | No | No | Yes | No | [27] |
| MXene-based aerogels | No | Yes | Yes | No | [28] |
| Ag NW/PVA | No | No | No | No | [29] |
| EVA@PPy | Yes | Yes | Yes | Yes | This work |

CNT_S_‒carbon nanotubes, PDMS‒polydimethylsiloxane, PDPU‒polysiloxanedimethylglyoxime-based polyurethane, PU‒polyurethane, PANI‒polyaniline, PVA‒polyvinylalcohol, EVA‒ethylene-co-vinyl acetate, PPy‒polypyrrole.

**References**

[1] P. Wang, J. Cao, Y. Li, G. Sun, H. Shao, C. Meng, *Chem. Eng. J.* **2024**, 495, 153525.

[2] S. Hoon Hong, T. Yeon Kim, S. Cheong, H. Bae, K. Hyun Yu, S. Kwang Hahn, *Chem. Eng. J.* **2023**, 476, 146559.

[3] J. Chen, J. Zhang, Z. Luo, J. Zhang, L. Li, Y. Su, X. Gao, Y. Li, W. Tang, C. Cao, Q. Liu, L. Wang, H. Li, *ACS Appl. Mater. Interfaces* **2020**, 12, 22200-22211.

[4] W. Wang, P. Guo, X. Liu, M. Chen, J. Li, Z. Hu, G. Li, Q. Chang, K. Shi, X. Wang, K. Lei, *Adv. Funct. Mater.* **2024**, 34, 2316346.

[5] J. Xiong, G. Thangavel, J. Wang, X. Zhou, P.S. Lee, *Sci. Adv.* **2020**, 6, eabb4246.

[6] H. Li, X. Hu, C. Li, Y. Sun, H. Jiang, R. Zhou, X. Wu, Y. Tang, X. Ding, *ACS Appl. Nano Mater.* **2023**, 6, 12095-12104.

[7] X. Peng, K. Dong, C. Ye, Y. Jiang, S. Zhai, R. Cheng, D. Liu, X. Gao, J. Wang, Z.L. Wang, *Sci. Adv.* 6, eaba9624.

[8] S. Si, C. Sun, J. Qiu, J. Liu, J. Yang, *Appl. Mater. Today* **2022**, 27, 101508.

[9] T. Liu, Z. Zhao, R. Liang, H. He, Y. Liu, K. Yu, M. Chi, B. Luo, J. Wang, S. Zhang, C. Cai, S. Wang, S. Nie, *Adv. Funct. Mater.* **2025**, 35, 2500207.

[10] L. Liu, J. Li, Z. Tian, X. Hu, H. Wu, X. Chen, L. Zhang, W. Ou-Yang, *Nano Energy* **2024**, 128, 109817.

[11] M. Jing, J. Zhou, P. Zhang, D. Hou, J. Shen, J. Tian, W. Chen, *ACS Appl. Mater. Interfaces* **2022**, 14, 55119-55129.

[12] W. Li, X. Jin, X. Han, Y. Li, W. Wang, T. Lin, Z. Zhu, *ACS Appl. Mater. Interfaces* **2021**, 13, 19211-19220.

[13] A. Huang, S. Gu, Z. Yang, X. Chen, M. He, X. Peng, **2025**, 63, 709-723.

[14] Z. Qiao, A. Wei, K. Wang, N. Luo, Z. Liu, *J. Alloys Compd.* **2022**, 917, 165503.

[15] L.-Q. Tao, K.-N. Zhang, H. Tian, Y. Liu, D.-Y. Wang, Y.-Q. Chen, Y. Yang, T.-L. Ren, *ACS Nano* **2017**, 11, 8790-8795.

[16] Z. Hu, Y. Wu, L. Tang, W. Zhou, J. Wang, W. Wu, M. Li, L. Wang, *Cellulose* **2025**, 32, 6433-6447.

[17] H. Chen, D. Guo, X. Lei, W. Wu, X. Guo, Y. Li, X. Weng, S. Liu, F. Liu, *ACS Appl. Mater. Interfaces* **2023**, 15, 21435-21443.

[18] C. Parameswaran, D. Gupta, *J. Mater. Chem. C* **2018**, 6, 5473-5481.

[19] K.-H. Ha, W. Zhang, H. Jang, S. Kang, L. Wang, P. Tan, H. Hwang, N. Lu, *Adv. Mater.* **2021**, 33, 2103320.

[20] W. Yang, Y. Liu, Z. Zhang, Q. Li, T. Yu, Y. Li, *Compos. Sci. Technol.* **2023**, 232, 109884.

[21] S. Liu, F. Yuan, M. Sang, J. Zhou, J. Zhang, S. Wang, J. Li, S. Xuan, X. Gong, *J. Mater. Chem. A* **2021**, 9, 6913-6923.

[22] J. Xiong, G. Thangavel, J. Wang, X. Zhou, P.S. Lee, *Sci. Adv.* 6, eabb4246.

[23] Y. Liu, Y. Zheng, Z. Wu, L. Zhang, W. Sun, T. Li, D. Wang, F. Zhou, *Nano Energy* **2021**, 79, 105422.

[24] S. Kim, M. Amjadi, T.-I. Lee, Y. Jeong, D. Kwon, M.S. Kim, K. Kim, T.-S. Kim, Y.S. Oh, I. Park, *ACS Appl. Mater. Interfaces* **2019**, 11, 23639-23648.

[25] J. Liao, X. Dai, J. Han, J. Yang, Y. Wu, Y. Cao, Y. Qiu, Y. Wang, L.-B. Huang, H. Ni, W. Feng, *Nano Energy* **2024**, 121, 109252.

[26] Z. Wang, R. Jiang, G. Li, Y. Chen, Z. Tang, Y. Wang, Z. Liu, H. Jiang, C. Zhi, *ACS Appl. Mater. Interfaces* **2017**, 9, 22685-22693.

[27] U. Pharino, Y. Sinsanong, S. Pongampai, T. Charoonsuk, P. Pakawanit, S. Sriphan, N. Vittayakorn, W. Vittayakorn, *Radiat. Phys. Chem.* **2021**, 189, 109720.

[28] Q. Sun, X. Zhang, P. Gu, X. Liang, Z. Hu, X. Yang, M. Liu, J. Huang, G. Zu, *Adv. Funct. Mater.* **2024**, 34, 2308537.

[29] P. Wang, G. Li, J. Liu, Z. Hou, C. Meng, S. Guo, C. Liu, S. Fan, *Adv. Mater. Interfaces* **2021**, 8, 2170110.
